# A Liquid to Solid Phase Transition Underlying Pathological Huntingtin Exon1 Aggregation

**DOI:** 10.1016/j.molcel.2018.04.007

**Published:** 2018-05-17

**Authors:** Thomas R. Peskett, Frédérique Rau, Jonathan O’Driscoll, Rickie Patani, Alan R. Lowe, Helen R. Saibil

**Affiliations:** 1 Institute of Structural and Molecular Biology, Birkbeck College and University College London, London, WC1E 7HX, UK; 2 Institute of Neurology, University College London, London, WC1N 3BG, UK; 3 The Francis Crick Institute, London, NW1 1AT, UK; 4 London Centre for Nanotechnology, University College London, London, WC1H 0AH, UK

**Keywords:** huntingtin exon1, aggregation, phase transition, polyQ, electron tomography, fluorescence microscopy

## Abstract

Huntington’s disease is caused by an abnormally long polyglutamine tract in the huntingtin protein. This leads to the generation and deposition of N-terminal exon1 fragments of the protein in intracellular aggregates. We combined electron tomography and quantitative fluorescence microscopy to analyze the structural and material properties of huntingtin exon1 assemblies in mammalian cells, in yeast, and *in vitro*. We found that huntingtin exon1 proteins can form reversible liquid-like assemblies, a process driven by huntingtin’s polyQ tract and proline-rich region. In cells and *in vitro*, the liquid-like assemblies converted to solid-like assemblies with a fibrillar structure. Intracellular phase transitions of polyglutamine proteins could play a role in initiating irreversible pathological aggregation.

## Highlights

• Aggregates of huntingtin exon1 exist in distinct liquid-like and solid-like forms
• Liquid-like assembly formation is driven by polyQ and proline-rich regions of exon1
• The liquid-like assemblies convert into solid-like assemblies *in vitro* and in cells
• Electron tomography reveals liquid and solid assemblies have distinct structures

## Graphical Abstract

**Figure:**
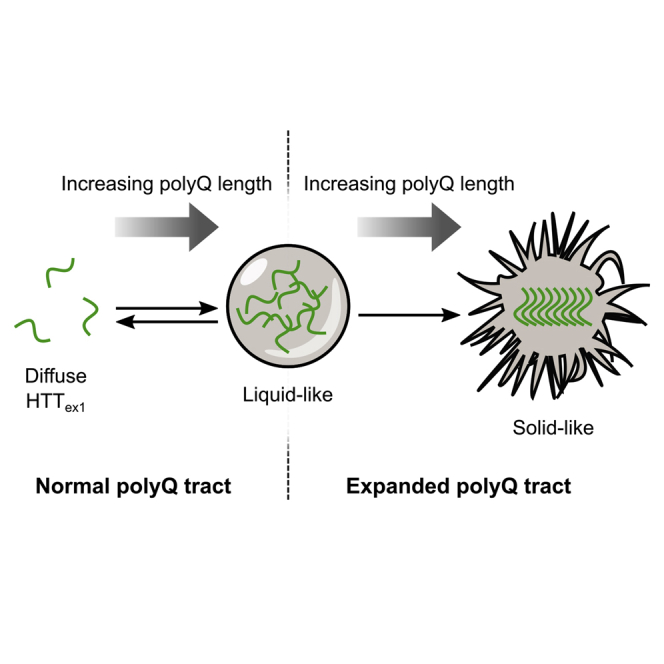

## Teaser

In Huntington’s disease, a polyglutamine expanded exon1 fragment of the huntingtin protein forms aggregates in the brains of affected individuals. Peskett et al. show that this protein fragment can form reversible liquid-like assemblies, which convert into solid-like fibrillar assemblies when the polyglutamine tract reaches disease-associated lengths.

## Introduction

Huntington’s disease (HD) is an incurable neurodegenerative disease, caused by a polyglutamine (polyQ) tract expansion in the huntingtin (HTT) protein (Bates et al., 2015). In humans, polyQ repeats ≥42 invariably cause HD, and longer repeats cause earlier onset (Finkbeiner, 2011). Although loss of HTT function may partly account for HD pathogenesis, it is known that small N-terminal, so called “exon1” fragments of HTT (HTT_ex1_), generated by aberrant splicing (Sathasivam et al., 2013) are key mediators of toxicity. HTT_ex1_ proteins comprise 17 N-terminal amino acids followed by the polyQ tract (varying lengths), a proline-rich region (38 residues), and 12 C-terminal residues. Expression of HTT_ex1_ proteins with expanded polyQ tracts causes HD-like symptoms in mice (Mangiarini et al., 1996) and is associated with toxicity in a range of other organisms, including yeast (Faber et al., 1999, Jackson et al., 1998, Meriin et al., 2002). In addition, HTT_ex1_ is highly aggregation prone and is a major constituent of fibrillar aggregates found in the brains of HD patients (DiFiglia et al., 1997, Schilling et al., 2007). Such protein aggregates are a common feature of neurodegenerative diseases including Alzheimer’s disease, Parkinson’s disease, and amyotrophic lateral sclerosis (ALS) (Dugger and Dickson, 2017). Aggregation of HTT_ex1_ is widely recapitulated in model systems and, like toxicity, is polyQ length dependent. Despite a clear link between HTT_ex1_ aggregation and toxicity, little is known about the aggregation mechanism in cells.

One possible aggregation mechanism is the classical nucleated growth model, whereby a critical nucleus, possibly a misfolded polyQ protein, initiates aggregation that proceeds by a “dock-lock” mechanism, with monomers adding to the growing fibril (Esler et al., 2000). This model can explain aggregation of simple polyQ peptides (Chen et al., 2002, Kar et al., 2011). In contrast, *in vitro* structural studies of HTT_ex1_ aggregation have identified small rounded oligomers, amorphous aggregates, and fibrils with various dimensions, suggesting a more complex mechanism (Crick et al., 2013, Legleiter et al., 2010, Poirier et al., 2002, Scherzinger et al., 1997, Wetzel, 2012). An alternative model proposes that amyloid nuclei initially form via intermediate higher-order assemblies such as oligomers (Lee et al., 2007, Vitalis and Pappu, 2011), an idea supported by *in vitro* biophysical experiments showing that oligomers appear in aggregation reactions prior to fibril formation (Crick et al., 2013, Jayaraman et al., 2012). In cells, biophysical and single-molecule experiments also provide evidence that HTT_ex1_ forms transient oligomers (Li et al., 2016, Ossato et al., 2010, Takahashi et al., 2007), though these are not seen consistently (Miller et al., 2011). Furthermore, these assemblies are not necessarily intermediates in the aggregation pathway, and off-pathway reaction products could be artifacts of *in vitro* systems. Thus, direct structural evidence of aggregation intermediates, particularly in the cell, is lacking.

Recent progress in understanding the formation of membrane-less compartments in cells, such as stress granules, raises another possible aggregation mechanism for HTT_ex1_. These compartments, whose components are often enriched in disordered regions with low sequence complexity (LC), appear to form by liquid-liquid demixing (Brangwynne et al., 2009, Kroschwald et al., 2015, Molliex et al., 2015). Within such phase-separated compartments, components are typically mobile and may exchange with the cytoplasm. However, liquid assemblies formed by the LC protein FUS may aberrantly convert into a solid-like state, and this is accelerated by mutations associated with ALS (Patel et al., 2015).

Although the aggregation mechanism of HTT_ex1_ *in vivo* is unclear, the end products of aggregation have been well characterized in cells. HTT_ex1_-fluorescent protein fusions commonly assemble into micron-sized aggregates, several orders of magnitude larger than the assemblies that are often studied *in vitro*. A limited number of ultrastructural studies suggest that these aggregates have fibrillar or granular substructures (DiFiglia et al., 1997, Legleiter et al., 2010, Scherzinger et al., 1997). Cryo-electron tomography experiments have recently confirmed the fibrillar structure of HTT_ex1_ aggregates (Bäuerlein et al., 2017). Using fluorescence microscopy, others have found that these aggregates can be morphologically and biophysically distinct (Caron et al., 2014, Dehay and Bertolotti, 2006, Duennwald et al., 2006a, Duennwald et al., 2006b). How are these different assemblies related to the aggregation mechanism?

In this study, we use a combination of correlative light and electron microscopy (CLEM) and time-lapse fluorescence microscopy to study the aggregation pathway across different spatial scales. We applied these techniques to mammalian, yeast, and *in vitro* models of HTT_ex1_ aggregation, to dissect the nanostructures, material properties, and aggregation pathway of HTT_ex1_ assemblies.

## Results

### Aggregation of HTT_ex1_ Proteins Can Involve a Conversion between Distinct Macroscopic Assemblies

**Figure 1:**
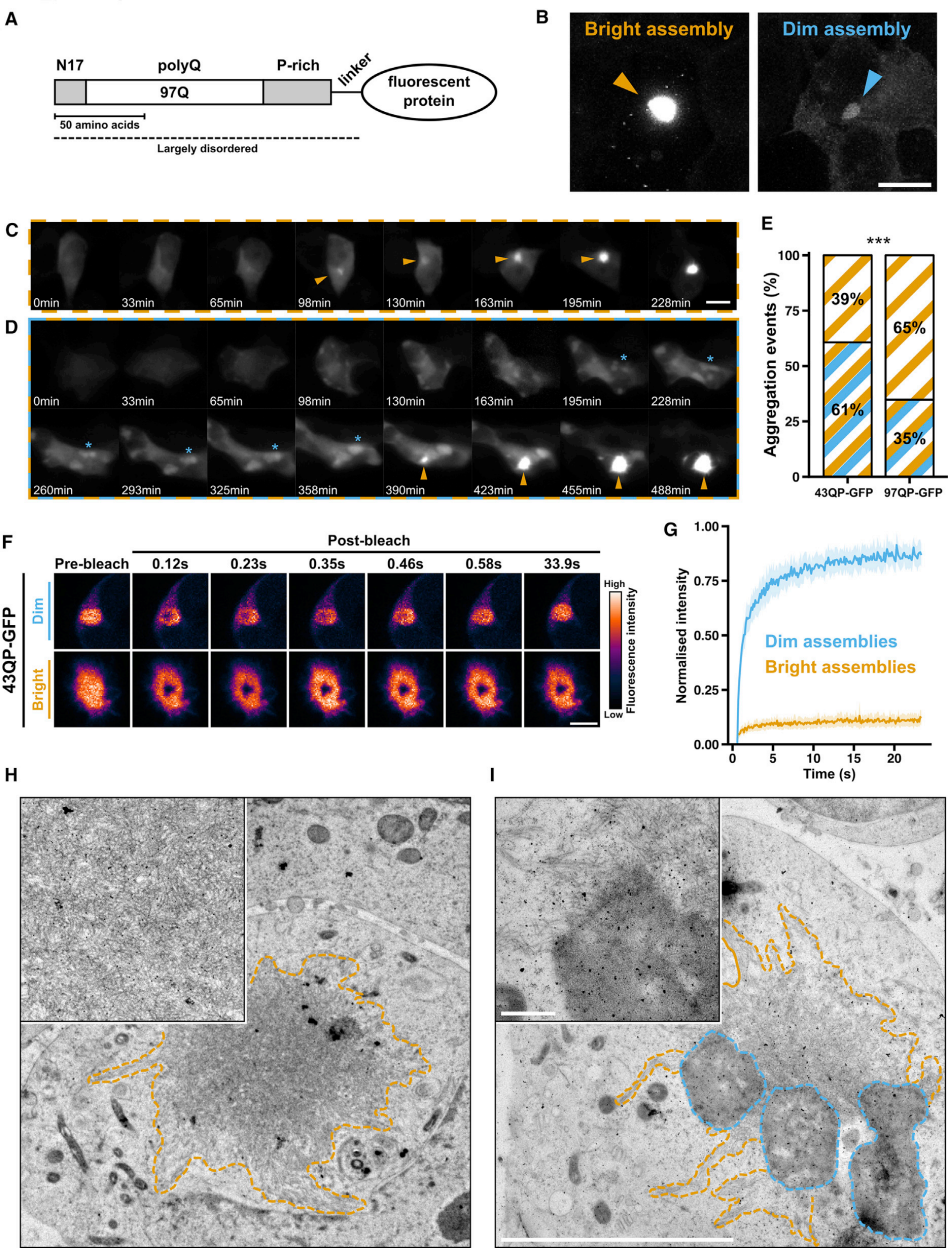
Aggregation of HTT_ex1_ Proteins Can Involve a Conversion between Distinct Macroscopic Assemblies (A) Domain organization of HTT_ex1_ constructs in this study. (B) Representative confocal maximum intensity projections of bright and dim 43QP-GFP assemblies. Scale bar, 10 μm. (C and D) Time-lapse fluorescence microscopy of 43QP-GFP aggregation without (C), and with (D), a visible intermediate dim assembly. Orange arrows: bright assembly formation. Blue asterisk: coalescence of dim assemblies. Scale bar, 10 μm. (E) Quantification of aggregation events occurring without (orange) and with (orange/blue) visible intermediate dim assemblies. n > 92 aggregation events per construct from three independent experiments. p = 0.0003, chi-square. (F) FRAP experiment showing high HTT_ex1_ mobility in dim assemblies but not in bright assemblies. Scale bar, 3 μm. (G) Averaged FRAP recovery curves. Shaded areas represent 95% confidence interval (CI). Dim assemblies estimated mobile fraction = 84%, 95% CI: 83%–85%, n = 20; bright assemblies estimated mobile fraction = 10%, 95% CI: 10%–11%, n = 20. See also Table S1. (H) EM projection image of a 43QP-GFP bright assembly (orange dashes). Higher-magnification image (inset) shows a network of fibrillar structures. (I) 43QP-GFP bright assembly (orange) and dim assemblies (blue). Higher-magnification image (inset) shows fibrillar structures emanating from the dim assemblies. Low-magnification scale bar, 5 μm; high-magnification scale bar, 500 nm.

To explore the aggregation pathway of polyQ-containing proteins, we induced expression of HTT_ex1_ proteins with different polyQ lengths (25, 43, or 97), fused to a C-terminal eGFP tag (Figure 1A) in HEK293 cells, and followed their expression by time-lapse fluorescence microscopy for 24–48 hr. We will refer to these proteins as 25, 43, or 97QP-GFP, where the number indicates the polyQ length (e.g., 97Q) and the P indicates the C-terminal proline-rich region of HTT_ex1_ (Figure 1A).

As previously observed, HTT_ex1_ aggregation was characterized by the rapid growth of an intensely fluorescent aggregate that sequestered the entire fluorescence signal in the cell (Figure 1C). We called these aggregates “bright assemblies” (Figure 1B). In some cases, we observed aggregation events whereby one or more weakly fluorescent structures appeared in the cell prior to the formation of a bright assembly. These structures, which we called “dim assemblies” (Figure 1B), could grow by coalescence (Figure 1D, blue asterisk) and exist for hours before a bright assembly grew from the edge of a dim assembly, sequestering all the fluorescence signal in the cell (Figure 1D, orange arrow; Video S1). We quantified these aggregation trajectories by computationally tracking single cells (Bove et al., 2017) and measuring their fluorescence intensity distributions over time, demonstrating that progression from diffuse fluorescence to a bright assembly, with or without an intermediate dim assembly, involves distinct changes in the fluorescence intensity distributions (Figures S1A and S1B).

We assigned the aggregation events in our time-lapse movies into one of two categories: formation of a bright assembly via a visible dim assembly, or formation of a bright assembly without a visible intermediate dim assembly. In 61% of 43QP-GFP aggregation events, we observed bright assemblies emerging via dim assemblies (Figure 1E). This reduced to 35% for 97QP-GFP. These experiments suggest that in some cases, HTT_ex1_ aggregation involves intermediate dim assemblies. Lengthening the polyQ tract may either reduce the formation of dim assemblies or accelerate their progression to bright assemblies. 25QP-GFP also formed dim assemblies, which could coalesce (Figure S1C), but these did not progress to bright assemblies (Figure S1D). This suggests that HTT_ex1_ can form dim assemblies with sub-toxic polyQ lengths but that progression to bright assemblies requires aberrant polyQ expansion. To test the effect of reduced HTT_ex1_ expression on assembly formation, we generated stable cell lines expressing 25QP-GFP or 97QP-GFP (see STAR Methods). Time course analysis confirmed that, as with transfected cells, dim assemblies formed with both polyQ lengths, whereas bright assemblies required polyQ expansion (Figure S1E).

Next we characterized the biophysical properties of dim and bright assemblies using fluorescence recovery after photobleaching (FRAP), which monitors the turnover of fluorescent molecules in a sub-cellular region bleached by a focused laser (Figure 1F). HTT_ex1_ was highly mobile within dim assemblies (estimated mobile fraction = 84%, with 95% confidence interval [CI]: 83%–85%, n = 20) and highly immobile in bright assemblies (estimated mobile fraction = 10%, 95% CI: 10%–11%, n = 20) (Figure 1G). Bright assemblies were also ∼20–30× brighter than dim assemblies (p < 0.0015; Figure S1F) and had spiky, irregular edges, whereas dim assemblies were more spherical in shape (Video S2; Figure S1G).

To investigate the nanoscale organization of these assemblies, we carried out correlative light and electron microscopy (CLEM) experiments on high-pressure frozen, freeze-substituted cells. Electron tomography of bright assemblies showed that they comprised a tightly interwoven meshwork of fibers with diameters around 13 nm (Figure 1H; Video S3), and their irregular shapes arose from bundles of fibers. The fluorescence signal of dim assemblies was difficult to detect in cell sections, likely due to the loss of signal associated with sample thinning. However, we made use of the observation that bright assemblies formed at the edges of dim assemblies to locate likely candidates for dim assemblies (Figure S2A). Dim assemblies had an amorphous, granular appearance (note that staining intensity does not necessarily correspond to protein concentration) and, like bright assemblies, were not enclosed by membranes (Figure 1I). Fibers from neighboring bright assemblies were seen partially inside or at the edges of dim assemblies (Figure 1I; Figure S2B).

Dim assemblies weakly colocalized with stress granules (Pearson coefficients: 0.20 ± 0.05 and 0.11 ± 0.05 with G3BP1 and TIA1 markers, respectively), which are also granular and internally mobile (Buchan and Parker, 2009, Souquere et al., 2009) (Figure S2C), suggesting partial overlap, but sequestration of HTT_ex1_ into stress granules cannot account for the existence of the dim assemblies.

Overall, our results suggest that in aggregation events involving a dim assembly, HTT_ex1_ proteins initially form amorphous, internally mobile assemblies and convert to fibrillar, internally immobile assemblies over a timescale of minutes to hours.

### Probing HTT_ex1_ Assembly States in Yeast

The budding yeast *Saccharomyces cerevisiae* has long been used as a model system to study HTT_ex1_ aggregation (Krobitsch and Lindquist, 2000, Mason and Giorgini, 2011). Despite its extensive use, the assemblies we observed in mammalian cells have not, to our knowledge, been described in yeast. To test the robustness of our observations, we therefore asked whether we could recapitulate the different forms of HTT_ex1_ assembly in yeast. Additionally yeast would allow us to probe the properties and structures of the assemblies more easily than in our mammalian cell system: yeast is well suited to medium-throughput CLEM studies of aggregation due to its excellent freezing properties and rate and synchronicity of cell growth (Kukulski et al., 2011).

**Figure 2:**
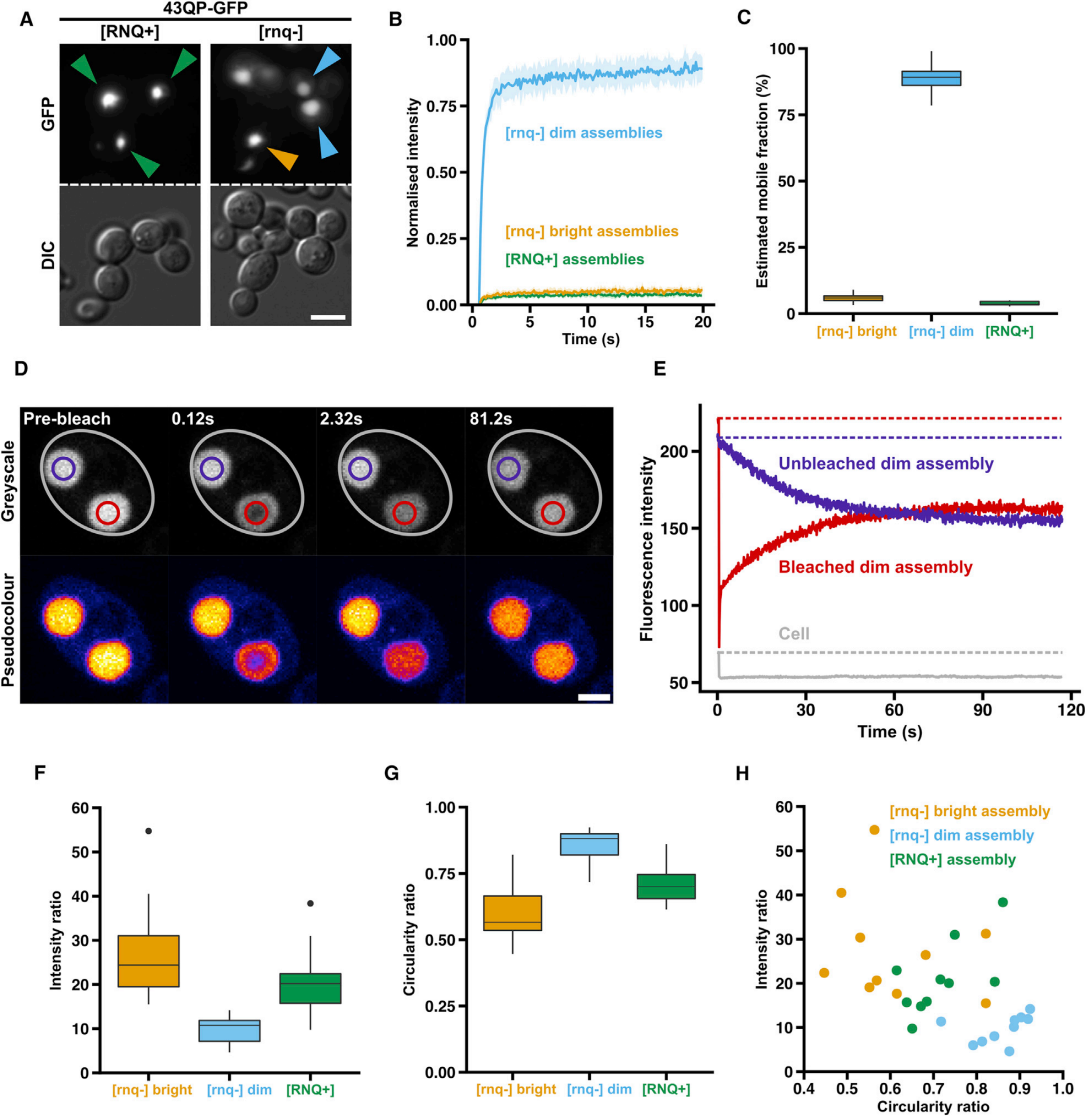
Probing HTT_ex1_ Assembly States in Yeast (A) Widefield microscopy of 43QP-GFP assemblies formed in [RNQ+] cells (green arrows), and [rnq−] cells (blue and orange arrows, showing dim and bright assemblies, respectively). Scale bar, 5 μm. (B) Averaged FRAP recovery curves. Shaded areas: 95% confidence intervals. See also Table S1. (C) Estimated mobile fractions of assemblies based on (B). Bright [rnq−] versus dim [rnq−] p = 6.3 × 10–13; bright [rnq−] versus [RNQ+] p = 0.003; dim [rnq−] versus [RNQ+] p = 3.2 × 10–12; Welch’s two-sample t test. (D) Photo bleaching of a dim assembly (red outline) in a yeast cell (gray outline) containing a second, coexisting dim assembly (purple outline). Scale bar, 3 μm. (E) Quantification of data in (D). Solid lines show mean fluorescence intensities in the corresponding colored regions in (D). Dotted lines highlight initial fluorescence levels in the corresponding colored regions. See also Table S2. (F) Intensity ratios of assemblies. Bright [rnq−] versus dim [rnq−] p = 0.0009; bright [rnq−] versus [RNQ+] p = 0.16; dim [rnq−] versus [RNQ+] p = 0.0019; Welch’s two sample t test. Black dots represent individual data points that fell outside the whiskers of the boxplots. (G) Circularity ratios of assemblies. Bright [rnq−] versus dim [rnq−] p = 0.0001; bright [rnq−] versus [RNQ+] p = 0.04; dim [rnq−] versus [RNQ+] p = 0.0006; Welch’s two-sample t test. (H) Plot of intensity ratios versus circularity ratios.

When we first expressed HTT_ex1_ proteins in yeast, we only observed intensely fluorescent assemblies, resembling the bright assemblies we had observed in mammalian cells. We wondered whether this might be due to the presence of the prion form of Rnq1 (denoted [RNQ+]) in our strains: although Rnq1 in its prion conformation is required for polyQ length-dependent toxicity in yeast (Meriin et al., 2002), mammals do not have an Rnq1 homolog. Given the fibrillar structures of bright assemblies in mammalian cells, and the observation that [RNQ+] is required for *de novo* formation of the prion form of the translation termination factor Sup35 (Derkatch et al., 2000), which forms fibrillar assemblies in yeast (Kawai-Noma et al., 2010, Tyedmers et al., 2010), we reasoned that [RNQ+] might favor formation of bright assemblies, making dim assemblies rare or very transient. When we expressed HTT_ex1_ constructs in yeast strains lacking the Rnq1 prion (denoted [rnq−]), we observed both intensely and weakly fluorescent assemblies, as we had done in mammalian cells, suggesting that HTT_ex1_ can form dim and bright assemblies in yeast (Figure 2A).

FRAP analysis of the yeast assemblies showed that HTT_ex1_ molecules were able to diffuse rapidly within dim assemblies but were immobile in bright assemblies, regardless of whether the bright assemblies had formed in [rnq−] or [RNQ+] cells (Figure 2B). The mobile fractions, estimated from the FRAP curves, were 89% (dim, [rnq−]), 6% (bright, [rnq−]) and 4% ([RNQ+]) (Figure 2C). This demonstrates that the two types of assembly have similar biophysical properties in yeast and mammalian cells. 97QP fused to the engineered monomeric fluorescent protein mEOS3.1 similarly formed both types of assembly (Figure S3A), suggesting that eGFP does not affect the nucleation and properties of the two phases.

To ask whether HTT_ex1_ could move between dim assemblies and the cytosol, we took advantage of cells containing two dim assemblies of approximately equal size (Figure 2D). Upon bleaching one of the two assemblies, the bleached area underwent a rapid partial recovery (∼2 s) but did not immediately return to its pre-bleached intensity (Figure 2E). On a slower timescale (∼2 min), the intensities of the two assemblies gradually equalized, while the average fluorescence intensity in the cell remained constant. Thus, HTT_ex1_ molecules can exchange between dim assemblies and the cytosol, but this occurs approximately 55× slower than exchange within the assemblies themselves (Table S2).

When bright assemblies formed in mammalian cells, they seemed to sequester all available HTT_ex1_, whereas dim assemblies could coexist with a pool of diffuse cytosolic HTT_ex1_. To compare HTT_ex1_ sequestration in the different assemblies in yeast, we measured the intensity ratios (IRs) of GFP fluorescence in the assemblies versus the cytosol, where IR = Intensity_assembly_/Intensity_cytosol_ (see STAR Methods). Bright assemblies were enriched in HTT_ex1_ (median IR of 24 and 20 for [rnq−] and [RNQ+] assemblies, respectively). In contrast, dim assemblies were less enriched (median IR = 11, Figure 2F).

As in mammalian cells, bright assemblies in yeast appeared to have less regular shapes than dim assemblies. The shapes of intracellular assemblies can often reflect underlying physical properties. For example, intracellular liquid-like assemblies tend to adopt spherical shapes due to their surface tension (Hyman et al., 2014). We therefore measured the circularity ratio (CR), where CR = 4π(Area/Perimeter^2^) (see STAR Methods), of the different assemblies (Figure 2G). Dim assemblies were more circular (median = 0.88) than bright assemblies (medians of 0.57 and 0.70 for [rnq−] and [RNQ+], respectively).

Plotting IR against CR for individual assemblies showed that dim assemblies cluster in the low IR, high CR region of the graph. Bright assemblies from [rnq−] and [RNQ+] backgrounds were found in the high IR, low CR region, but did not form separate clusters (Figure 2H). Thus, the distinct clusters were in agreement with our initial visual classification.

These data suggest that in the absence of the prion form of Rnq1, HTT_ex1_ proteins are capable of forming two types of higher-order assembly in yeast cells, which have biophysical and morphological properties analogous to the assemblies that we characterized in mammalian cells.

### Dim Assemblies Display Liquid-like Properties in Cells

**Figure 3:**
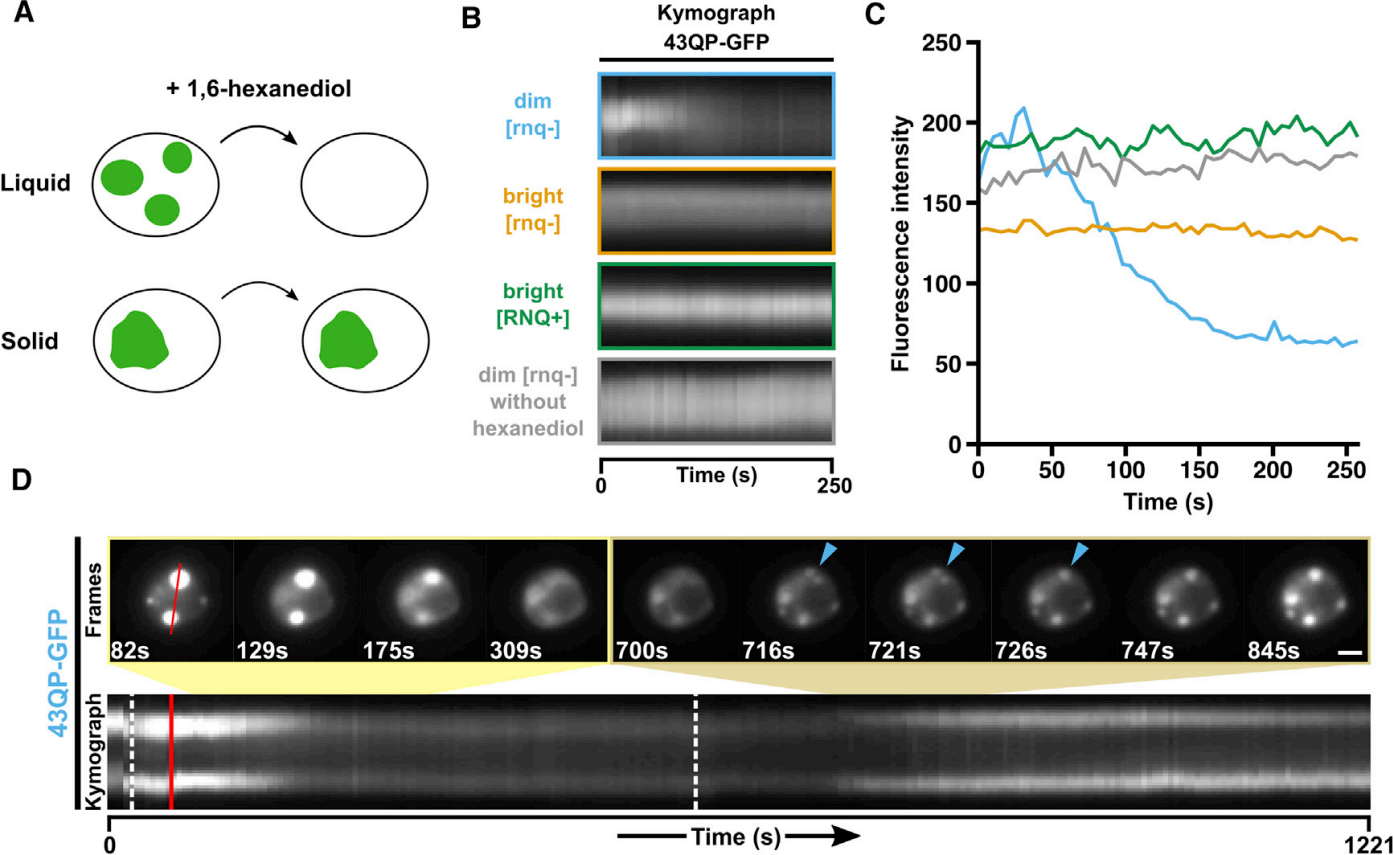
Dim Assemblies Display Liquid-like Properties in Cells (A) Addition of 1,6-hexanediol to digitonin-permeabilised cells can discriminate between liquid-like assemblies, which dissolve, and solid-like assemblies, which do not (Kroschwald et al., 2015). (B) Kymographs showing the effect of hexanediol addition on fluorescence intensities of HTT_ex1_ assemblies in digitonin-permeabilized yeast cells. Hexanediol was added at time = 0 s. (C) Quantification of fluorescence intensities in kymographs in (B). (D) Snapshots and kymograph of hexanediol addition (first dotted white line) to a yeast cell containing dim assemblies, followed by hexanediol removal (second dotted white line). The red line indicates the slice through which the kymograph was plotted. Blue arrow highlights coalescence. Images were linearly scaled to increase the visibility of the merging assemblies, saturating some of the pixels in the early frames. Note that dim assemblies do not sequester all the cytoplasmic fluorescence, which is visible throughout the experiment. Scale bar, 3 μm. Related to Video S4.

We sought to explore the nature of the chemical interactions underlying dim assemblies in yeast cells. 1,6-hexanediol is an aliphatic alcohol that has previously been used to study the weak hydrophobic interactions between FG-repeat containing nucleoporins, and to differentiate between liquid-like and solid-like membrane-less compartments (Figure 3A) (Kroschwald et al., 2015, Patel et al., 2007, Ribbeck and Görlich, 2002).

When hexanediol was added to dim assemblies, they dissolved within a few minutes, on a timescale comparable with the dissolution of liquid-like compartments under similar conditions (Figures 3B and 3C, blue; Figure S3B) (Kroschwald et al., 2015). In contrast, bright assemblies remained intact, irrespective of Rnq1 prion status (Figures 3B and 3C, orange and green; Figure S3B). Permeabilization of cells in the absence of hexanediol did not affect the integrity of dim assemblies (Figures 3B and 3C, gray; Figure S3B). We next asked whether removing hexanediol could reverse disassembly. When hexanediol was washed away, dim assemblies rapidly reformed, on timescales similar to their dissolution (Figure 3D; Video S4), and during reformation we noticed the coalescence of smaller assemblies into larger assemblies (Figure 3D, blue arrows).

This suggests that dim assemblies are highly reversible structures, maintained by weak hydrophobic interactions, whereas bright assemblies are maintained by stronger, possibly amyloid-like, interactions. In addition, the rapid reversibility, circularity, coalescence, and internal mobility of dim assemblies are consistent with liquid-like properties. From this point on, we therefore refer to dim and bright assemblies as liquid-like assemblies (LAs) and solid-like assemblies (SAs), respectively.

### Liquid-like and Solid-like HTT_ex1_ Assemblies Have Different Nanostructures

**Figure 4:**
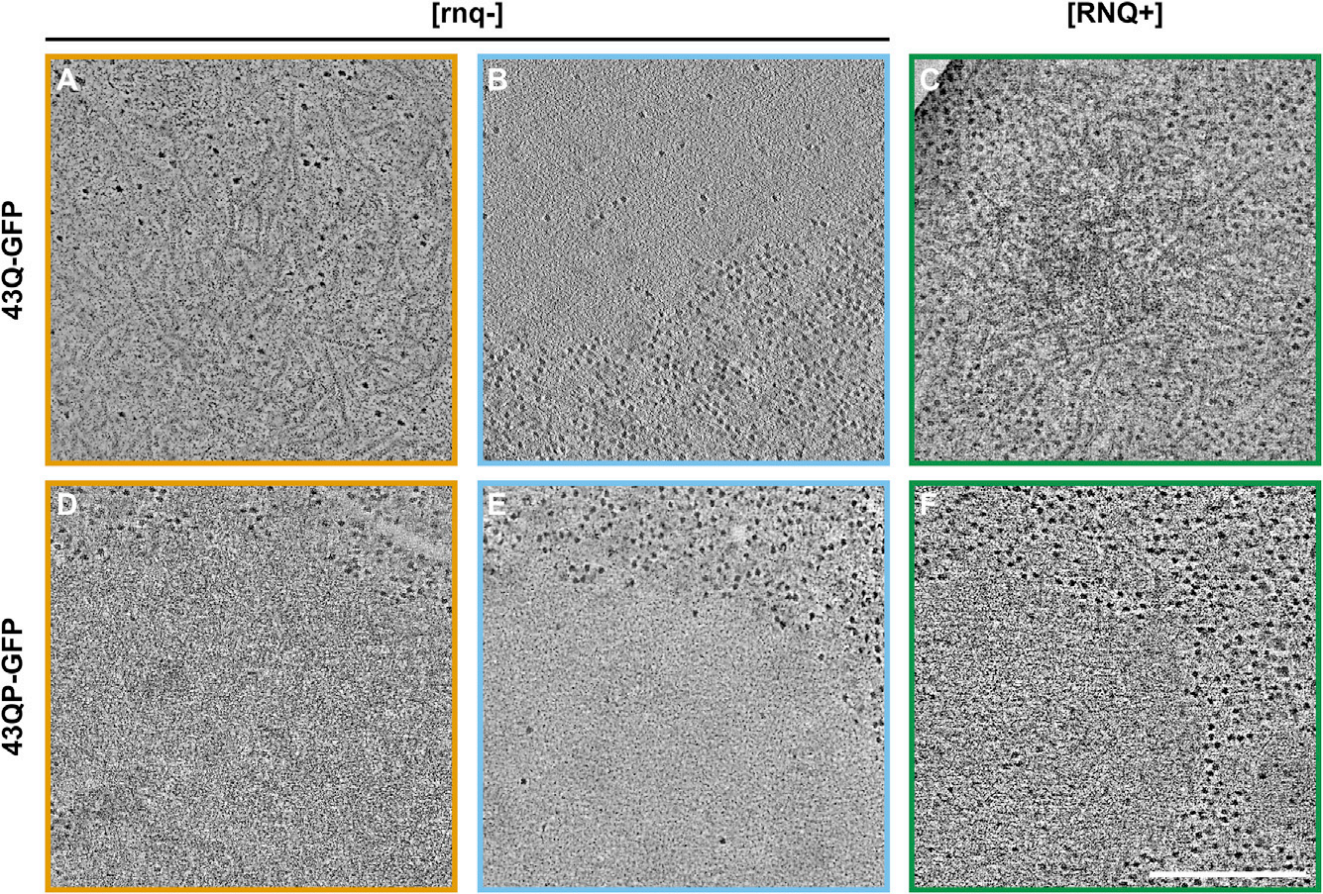
Liquid-like and Solid-like HTT_ex1_ Assemblies Have Different Nanostructures Electron tomography of yeast cells expressing 43Q-GFP and 43QP-GFP. Panels show slices through tomograms of the HTT_ex1_ assemblies. Colors indicate the type of assembly and prion status of the cells: orange, [rnq−] SAs; blue, [rnq−] LAs; green, [RNQ+] SAs. (A) 43Q-GFP SA in a [rnq−] cell, showing a fibrillar nanostructure. (B) 43Q-GFP LA in a [rnq−] cell, with a characteristic “smooth” appearance. (C) 43Q-GFP SA in a [RNQ+] cell. (D) 43QP-GFP SA in a [rnq−] cell. (E) 43QP-GFP LA in a [rnq−] cell. (F) 43QP-GFP SA in a [RNQ+] cell. Scale bar, 200 nm.

To characterize the structures of LAs and SAs in yeast cells, we carried out CLEM experiments, focusing on two constructs: 43Q-GFP and 43QP-GFP. 43Q-GFP SAs were composed of fibers with diameters of approximately 13 nm, consistent with the fibers that we had observed in mammalian cells (Figure 4A). In contrast, 43Q-GFP LAs appeared as “smooth,” membrane-less masses with no obvious substructure that largely excluded ribosomes (Figure 4B). Assemblies formed in [RNQ+] cells were always composed of fibers, in agreement with our observations in [rnq−] cells (Figure 4C).

SAs of 43QP-GFP, in both [rnq−] and [RNQ+] backgrounds, appeared to have a “granular” substructure with evidence of short fibrillar structures within the mass of the main assembly (Figures 4D and 4F). They therefore differed slightly to the obviously fibrillar assemblies of 43QP-GFP seen in mammalian cells, and of 43Q-GFP seen in yeast. However, LAs of 43QP-GFP had the characteristic appearance of LAs formed by other HTT_ex1_ proteins that we characterized (Figure 4E; Figure S4). The assembly-cytosol boundaries of LAs, but not SAs, were clearly demarcated, which was consistent with our circularity measurements.

Collectively, these data suggest that SAs have complex nanostructures that are indicative of the strong intermolecular interactions associated with amyloid deposits, whereas LAs appear to lack such structures.

### HTT_ex1_ Sequence Affects Assembly Formation

**Figure 5:**
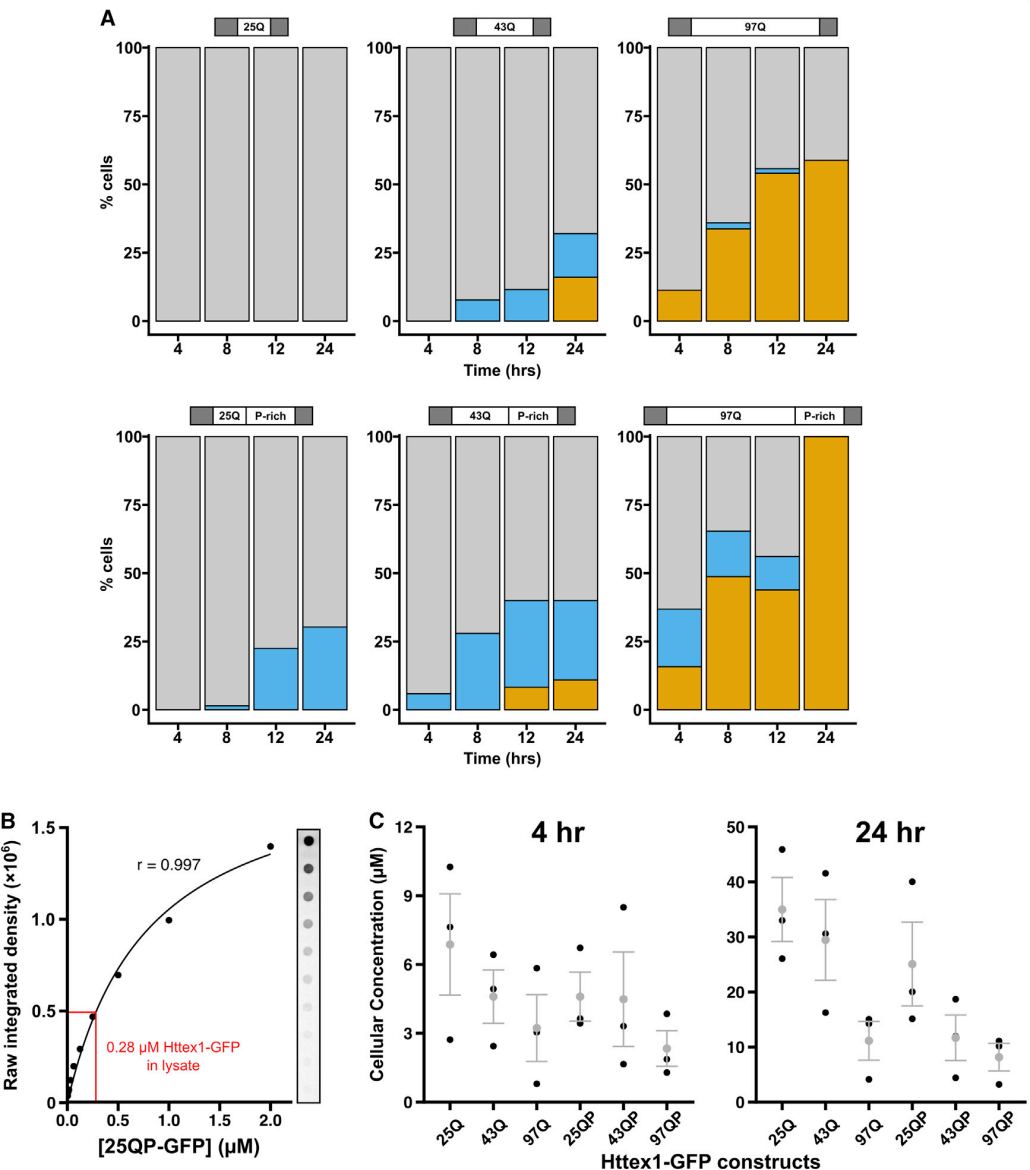
HTT_ex1_ Sequence Affects Assembly Formation (A) Yeast cells in the [rnq−] background expressing HTT_ex1_ constructs with different polyQ lengths, with or without the P-rich region, were imaged by widefield fluorescence microscopy at specific time points after induction of HTT_ex1_ expression. The percentage of cells containing an SA (orange), an LA (blue), or diffuse fluorescence (gray) was determined at each time point by manual counting. n = 207–259 cells per construct. (B) Quantitative dot blots calibrated with purified 25QP-GFP were used to determine HTT_ex1_ concentration in cellular lysates (red). (C) Cellular concentrations of HTT_ex1_ constructs at 4 and 24 hr. Plots show individual experiments (black) and mean ± SEM (gray).

The sequences of HTT_ex1_ proteins determine their aggregation propensities and toxicity (Dehay and Bertolotti, 2006, Duennwald et al., 2006b). However, how these effects are related to the structural properties of HTT_ex1_ assemblies is not well understood. We therefore compared a series of HTT_ex1_ constructs with different polyQ lengths, with or without the proline-rich (P-rich) region, to determine the effects of HTT_ex1_ sequence on the formation of LAs and SAs in yeast. We quantified the fraction of cells containing (1) an LA, (2) an SA, and (3) no assembly (diffuse fluorescence), at specific time points after expression induction (Figure 5A). We did not observe coexisting LAs and SAs in the same yeast cell.

In cells expressing 25Q-GFP, we did not observe assemblies, indicating that a 25Q tract is not sufficient to form macroscopic assemblies. However, 25QP-GFP began to form LAs at 8–12 hr, and by 24 hr, 25% of cells contained LAs, suggesting that the P-rich region can promote the formation of LAs. In our experiments, 25QP-GFP did not form SAs. 43Q-GFP was able to form both LAs and SAs, suggesting that lengthening the polyQ tract is sufficient to drive assembly formation, even in the absence of the P-rich region. In agreement with the idea that the P-rich region facilitates LA formation, 43QP-GFP formed LAs more readily than 43Q-GFP. As polyQ length was increased further (97Q-GFP and 97QP-GFP), SAs formed earlier and were more abundant than LAs. In cells expressing 43Q-GFP and 43QP-GFP, LAs formed earlier than SAs. With 97Q-GFP and 97QP-GFP, LAs were seen early on, but by 24 hr all assemblies were SAs, suggesting that LAs could convert to SAs, as seen in mammalian cells.

Expression levels were similar between different constructs and dropped slightly for longer constructs (Figure S5A), as expected for long polyQ tracts (Duennwald, 2011). To explore the concentration dependence of the assemblies we used quantitative dot blotting (see STAR Methods) to estimate intracellular HTT_ex1_ concentrations at 4 and 24 hr time points (Figures 5B and 5C). At 4 hr, when polyQ-expanded proteins began to form assemblies, HTT_ex1_ concentrations were in an ∼1–10 μM range, increasing to 3–46 μM at 24 hr, when 25QP-GFP (mean conc. 25 μM) had formed LAs in >25% of cells.

These data support the notion that both the polyQ tract and the P-rich region play important roles in driving the formation of higher-order assemblies of HTT_ex1_, as suggested previously (Crick et al., 2013, Dehay and Bertolotti, 2006, Duennwald et al., 2006b). In addition, the data demonstrate how the polyQ tract and P-rich region can affect the propensity of HTT_ex1_ to form assemblies with strikingly different biophysical properties. The observation that LAs appeared in cell populations before SAs, coupled to the fact that we did not observe coexisting LAs and SAs, suggests that LAs are on pathway to SAs. Moreover, increasing polyQ length to disease-associated lengths appears to accelerate this pathway.

### HTT_ex1_ Forms Liquid-like Assemblies by Liquid-Liquid Phase Separation

**Figure 6:**
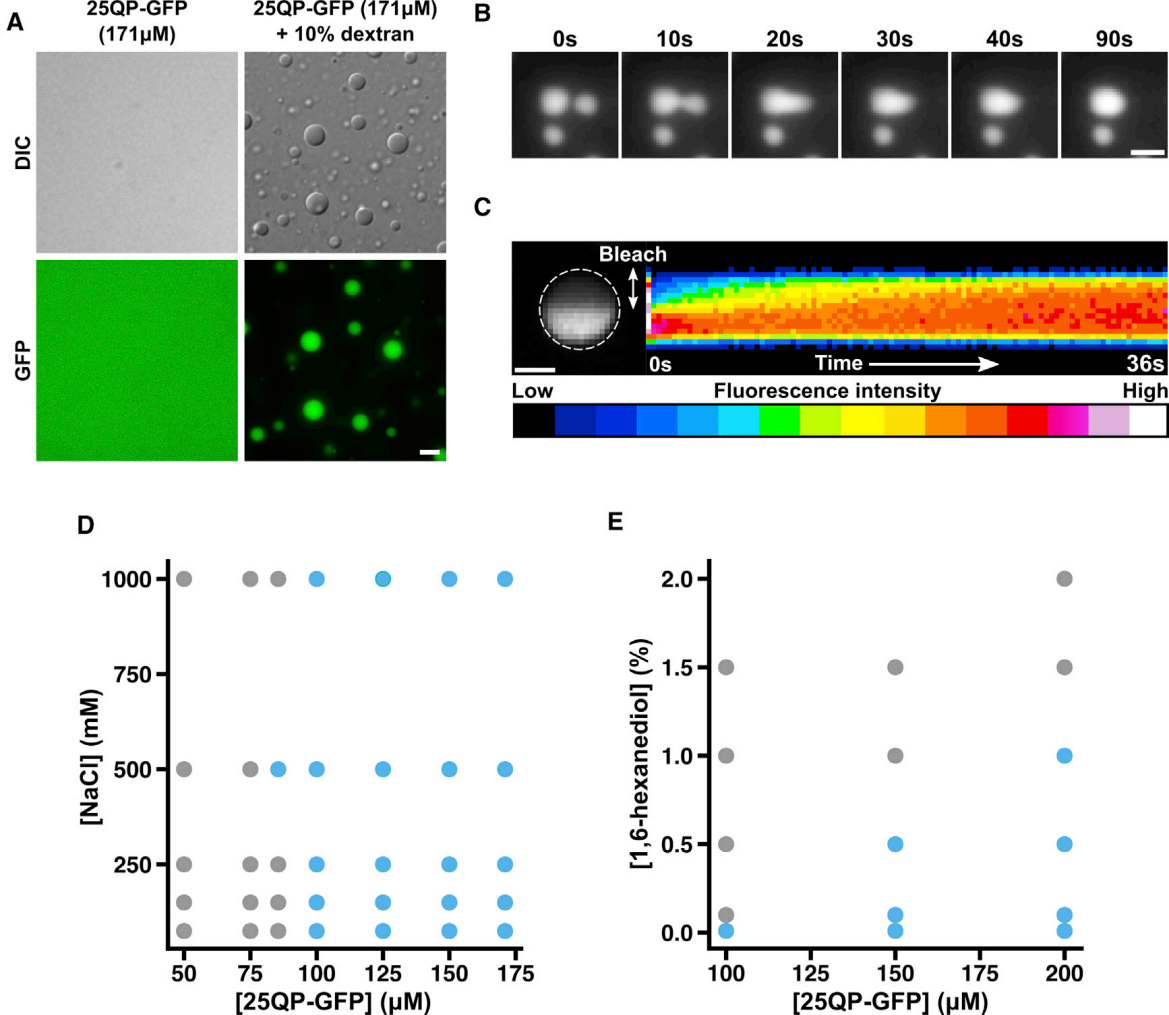
HTT_ex1_ Forms Liquid-like Assemblies by Liquid-Liquid Phase Separation (A) Liquid-liquid phase separation of 25QP-GFP induced by molecular crowding. Scale bar, 5 μm. (B) Fusion of 25QP-GFP droplets. Scale bar, 2 μm. See also Video S5. (C) Half-bleach (Brangwynne et al., 2009) of a 25QP-GFP droplet (white dashed outline). Kymograph shows redistribution of 25QP-GFP after the bleach. Scale bar, 1 μm. (D and E) Intermolecular interactions of 25QP-GFP in droplets. (D) Droplet formation at different salt and protein concentrations. The phase diagram indicates conditions where 25QP-GFP forms droplets (blue dots) and where it does not (gray dots). (E) Phase diagram showing the effect of 1,6-hexanediol concentrations on droplet formation. All experiments were carried out in the presence of 10% dextran as a crowding agent, and each condition was assessed at least twice.

How is assembly formation initiated? What is the relationship between LAs and SAs? To answer these questions, we reconstituted assembly formation *in vitro*, using recombinantly expressed and purified 25QP-GFP. Purified 25QP-GFP had the advantage of being more stable than other proteins with longer polyQ tracts. On its own, 25QP-GFP showed a diffuse distribution. In the presence of 10% dextran, which can be used to mimic crowding conditions in the cell (Patel et al., 2015), 25QP-GFP assembled into micron-sized, spherical droplets (Figures 6A; Figures S6A and S6B). BSA did not form droplets under the same conditions (Figure S6C). Smaller droplets of 25QP-GFP fused to form larger droplets, relaxing back into spherical shapes (Figure 6B; Video S5). We next asked whether 25QP-GFP molecules could diffuse within the droplets, using FRAP (Brangwynne et al., 2009). After bleaching, the fluorescence intensity in the unbleached region gradually decreased, while the intensity in the bleached region gradually increased, indicating mixing of 25QP-GFP within the droplets (Figure 6C). Our experiments demonstrate that 25QP-GFP forms liquid-like assemblies *in vitro*. We attempted to purify 43QP-GFP but it was highly aggregation-prone *in vitro*. Nevertheless, 43QP-GFP also showed signs of assembling into droplets at lower concentrations than 25QP-GFP and in the absence of crowding agent (Figure S6D), consistent with the idea that polyQ expansion promotes HTT_ex1_ assembly/aggregation.

Phase separation often involves electrostatic interactions to promote droplet formation (Elbaum-Garfinkle et al., 2015, Nott et al., 2015). We asked whether this was true for 25QP-GFP by assessing phase separation at different protein and salt concentrations (Figure 6D). Salt concentration had little effect on droplet formation, even at very high concentrations (up to 1 M), suggesting that electrostatic effects do not play a significant role in the formation and stability of 25QP-GFP droplets. Next, we tested the effect of increasing 1,6-hexanediol concentrations on droplet formation (Figure 6E). Hexanediol had a major destabilizing effect on droplets and prevented their formation even at very low concentrations (0.1%). Our data suggest that HTT_ex1_ can form macroscopic reversible assemblies by a liquid-liquid phase separation mechanism, likely due to weak hydrophobic interactions mediated by the polyQ and P-rich regions.

### Liquid-like HTT_ex1_ Assemblies Convert into Solid-like Assemblies

**Figure 7:**
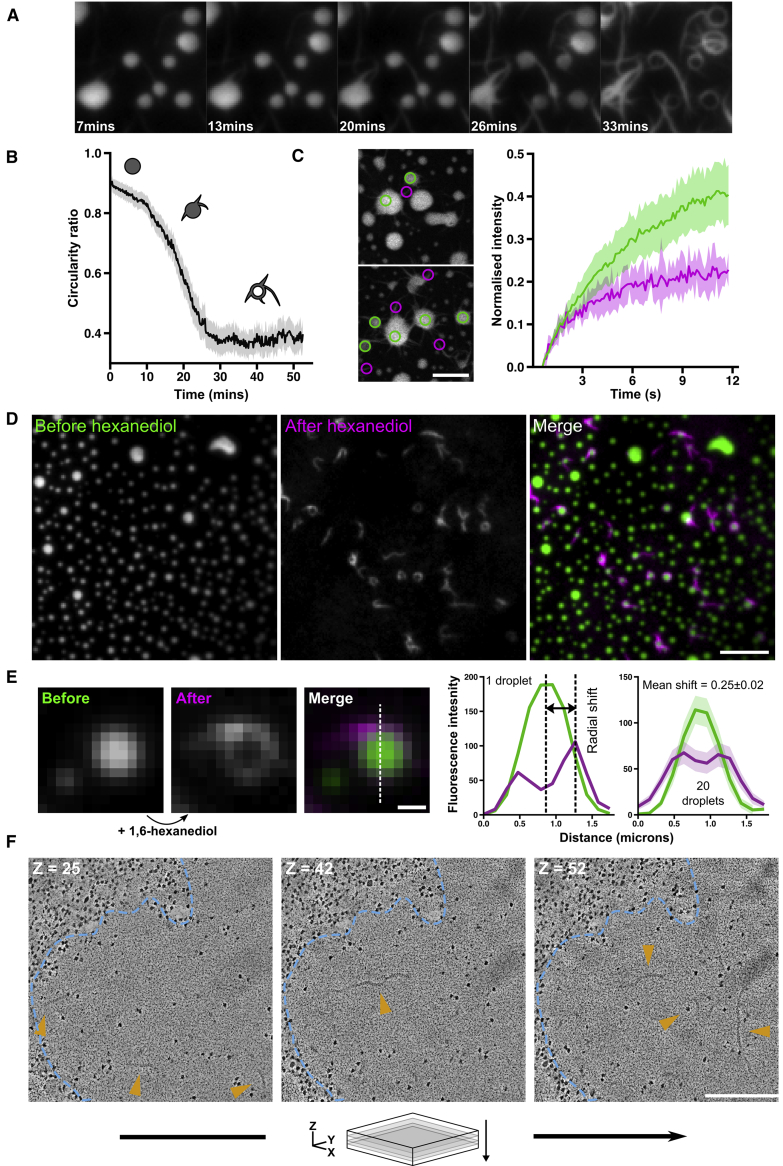
Liquid-like HTT_ex1_ Assemblies Convert into Solid-like Assemblies (A) Liquid-like droplets gradually convert into solid-like structures. Scale bar, 2 μm. (B) Quantification of the mean circularity of droplets during the conversion shown in (A). (C) FRAP of partially converted droplets comparing recovery of spike features (magenta) and droplet centers (green). Shaded region of graph represents 95% confidence interval. Scale bar, 2 μm. See also Table S1. (D) Droplets before (green) and after (magenta) hexanediol addition. Scale bar, 5 μm. (E) Quantification of early stages of droplet conversion (related to Figure 7D). Radial shift is the distance between intensity maxima in droplet linescans before (green) and after (magenta) hexanediol. n = 20 similarly sized droplets. Shaded regions: 95% confidence intervals. Scale bar, 0.5 μm. (F) Slices through a tomogram of a yeast cell containing a 43Q-GFP liquid-like assembly (blue dotted line) containing isolated fibrillar structures (orange arrows). Scale bar, 200 nm.

When imaging droplets of 25QP-GFP, we noticed that the droplets gradually developed irregularly shaped edges. To examine this phenomenon in more detail, we performed time-lapse total internal reflection fluorescence (TIRF) microscopy of 25QP-GFP droplets. Over a period of 30–60 min, macroscopic, spike-like projections began to grow from the edges of droplets, depleting the protein in the droplet centers so that they appeared hollow, and leading to a reduction in the average circularity of objects in the field of view (Figures 7A and 7B; Video S6). This suggests that phase-separated 25QP-GFP liquid-like droplets progressively convert into solid-like aggregates. After photo-bleaching, fluorescence recovery in the spike features at the edges of droplets was slower than recovery at the centers of the droplets, indicating reduced mobility of 25QP-GFP in the spikes (Figure 7C). This implies that the growth of spikes represents conversion of the reversible liquid-like state to an irreversible solid-like state, analogous to the phase transition of G156E FUS-GFP *in vitro* (Patel et al., 2015).

To distinguish the liquid-like from the solid-like components of the reaction and study their localization in droplets, we incubated droplets for 20 min to allow conversion to begin, then added hexanediol to dissolve the liquid-like components, leaving the solid-like components intact (Video S7). By imaging a fixed field of view before and after hexanediol addition, we could see that early solid-like structures localized to the edges of droplets, often as rings that were not visible before hexanediol addition, due to masking by the fluorescence of the droplets (Figure 7D). We quantified the position of these structures in droplets early during the conversion by measuring the radial shift in droplet maximum fluorescence intensity upon hexanediol addition (Figure 7E). Droplets with an ∼1 μm diameter had a mean radial shift of 0.25 ± 0.02 μm, suggesting that conversion begins in droplets, near droplet edges. We therefore looked for evidence of conversion from a liquid- to a solid-like state in yeast assemblies using FRAP. Occasionally we observed cells containing LAs that appeared to be undergoing a conversion similar to the one we had observed *in vitro*, with irregular features visible at the edge of the LA (Figure S7). FRAP revealed decreased mobility of HTT_ex1_ in these regions, suggesting a liquid- to solid-like conversion. To further explore the possibility of conversion in cells, we used electron tomography. We found isolated fibers buried within the featureless masses of some LAs, suggesting that irreversible solid-like structures can originate from reversible liquid-like assemblies in the cell as well as *in vitro* (Figure 7F). Such snapshots may represent the earliest stages in conversion, with fibers moving out toward the edges later on.

## Discussion

By applying CLEM and fluorescence microscopy to different HTT_ex1_ aggregation models, we found that HTT_ex1_ proteins can assemble into macroscopic liquid-like structures. In contrast to amyloid-like aggregates, these assemblies are maintained by weak hydrophobic interactions that are easily reversed by hexanediol. Our CLEM experiments show that liquid-like assemblies lack a visible substructure at the nanoscale, consistent with their high internal mobility assessed using FRAP. Over time, in cells and *in vitro*, the liquid-like assemblies gradually convert into assemblies that behave like solids. These solid-like assemblies are highly structured at the nanoscale and resemble aggregates that have been found in the brain tissue of HD patients (DiFiglia et al., 1997).

We propose an aggregation model whereby diffusely distributed HTT_ex1_ initially forms liquid-like assemblies by a liquid-liquid phase separation mechanism that is promoted by increasing HTT_ex1_ concentration and mediated by weak hydrophobic interactions between the polyQ and P-rich regions. Disease-associated polyQ expansions increase the propensity to form liquid-like assemblies, likely by lowering the critical concentration for phase separation. Formation of macroscopic liquid-like assemblies promotes a liquid to solid phase transition of HTT_ex1_ that is concomitant with the formation of an ordered, fibrillar nanostructure. Our aggregation model is consistent with the kinetics of multi-step aggregation models that propose metastable intermediates like micelles or oligomers (Ossato et al., 2010, Thakur et al., 2009) and may explain previous observations of HTT_ex1_ assemblies with different biophysical properties (Caron et al., 2014). As proposed previously for FUS, liquid-like assemblies could facilitate the assembly of more stable structures, by lowering the free-energy barrier of nucleation (Patel et al., 2015, ten Wolde and Frenkel, 1997).

In cells, HTT_ex1_ with sub-toxic polyQ lengths can form liquid-like assemblies but these do not convert to irreversible solid-like assemblies. The conversion occurs only when polyQ length extends beyond the threshold for HD. Yet, *in vitro*, 25QP-GFP droplets do convert to irreversible solid-like structures. Other proteins such as chaperones may influence the conversion process *in vivo*. Whether full-length HTT forms liquid-like assemblies in cells is also yet to be tested. Sequence analysis shows that the polyQ tract may play a role in HTT function (Tartari et al., 2008) but that role is unclear (Bates et al., 2015). HTT is associated with dynamic cytosolic compartments (Maiuri et al., 2017, Nath et al., 2015, Savas et al., 2008), and bioinformatic analyses suggest that polyQ tracts stabilize protein-protein interactions (Savas et al., 2008, Schaefer et al., 2012). It is tempting to speculate that HTT makes use of its “sticky” exon1 region to promote liquid-liquid phase separation.

The nature of the toxic species in HD is an area of intense research. Several studies point toward the protective effect of aggregates (Kuemmerle et al., 1999, Arrasate et al., 2004, Slow et al., 2005), yet others suggest that aggregates themselves potentiate toxicity (Li et al., 2016, Michalik and Van Broeckhoven, 2003, Woerner et al., 2016). Evidence from yeast indicates that toxicity depends on the proteins they sequester (Gong et al., 2012). As liquid-like aggregation intermediates are more amenable to chemical intervention than their solid counterparts, they might provide a means to promote or hinder aggregation, to ameliorate toxicity. HTT_ex1_ toxicity could also be probed by modulating aggregation via the liquid-like phase.

Recent studies show that phase-separated low complexity proteins can mature into more stable structures *in vitro* (Molliex et al., 2015, Patel et al., 2015, Zhang et al., 2015), and it has been hypothesized that similar changes in cellular assemblies, such as P-bodies and stress granules could contribute to neurodegenerative diseases (Li et al., 2013, Ramaswami et al., 2013). Our results provide a dramatic example of how HTT_ex1_ can aggregate via a liquid to solid conversion, and provide direct structural evidence that such conversions can take place in the cellular context.

## STAR★Methods

### Key Resources Table

**Table:** 

REAGENT or RESOURCE	SOURCE	IDENTIFIER
**Antibodies**		

Anti-GFP	Clontech Laboratories	Cat# 632381, RRID: AB_2313808
Anti-TIA-1	Santa Cruz Biotechnology	Cat# sc-1751, RRID: AB_2201433
Anti-G3BP	BD Biosciences	Cat# 611126, RRID: AB_398437
Goat anti-Mouse IgG (H+L) Secondary Antibody, DyLight 488	Thermo Fisher Scientific	Cat# 35502, RRID: AB_844397

**Bacterial and Virus Strains**		

Rosetta DE3 competent cells	Merck Millipore	70954

**Chemicals, Peptides, and Recombinant Proteins**		

99% 1,6-hexanediol	Sigma-Aldrich	240117
Dextran from *Leuconostoc mesenteroides* (average mol wt 64-76k)	Sigma-Aldrich	D8821
25QP-GFP	This study	N/A
43QP-GFP	This study	N/A

**Experimental Models: Cell Lines**		

HEK293	ATCC	CRL-1573
Flp-In 293	Thermo Fisher Scientific	R75007
Flp-In 293 - 25QP-GFP	This study	N/A
Flp-In 293 - 97QP-GFP	This study	N/A

**Experimental Models: Organisms/Strains**		

*S. cerevisiae*: Strain background: 74-D694	Chernoff et al., 1995	N/A

**Recombinant DNA**		

Plasmid: p425GAL1 (yeast)	Mumberg et al., 1995	N/A
Plasmid: 97QP-GFP (yeast)	This study	N/A
Plasmid: 97Q-GFP (yeast)	This study	N/A
Plasmid: 43QP-GFP (yeast)	This study	N/A
Plasmid: 43Q-GFP (yeast)	This study	N/A
Plasmid: 25QP-GFP (yeast)	This study	N/A
Plasmid: 97QP-mEOS3.1 (yeast)	This study	N/A
Plasmid: 25Q-GFP (yeast)	This study	N/A
Plasmid: 97QP-GFP (mammalian)	This study	N/A
Plasmid: 43QP-GFP (mammalian)	This study	N/A
Plasmid: 25QP-GFP (mammalian)	This study	N/A
Plasmid: pET His6-MBP-Asn10 TEV LIC vector	Scott Gradia	Addgene 29654

**Software and Algorithms**		

MATLAB	N/A	https://www.mathworks.com/products/matlab.html
Python	N/A	https://www.python.org/
Fiji	Schindelin et al., 2012	https://fiji.sc/
R	N/A	https://www.r-project.org/
Bayesian tracking library	Bove et al., 2017	https://github.com/quantumjot/BayesianTracker
TensorFlow	Abadi et al., 2016	https://www.tensorflow.org/
IMOD	Kremer et al., 1996	http://bio3d.colorado.edu/imod/
SerialEM	Mastronarde, 2005	http://bio3d.colorado.edu/SerialEM/

**Other**		

Dot blot device	Bio-Rad Laboratories	1706545
35mm Dish, No. 1.5 high tolerance coverslip, 14mm Glass diameter	Mattek	P35G-0.170-14-C
Micron-slide VI 0.4	Ibidi	80601
Serial connector for Micron-Slides	Ibidi	10830
Finder grids	Agar Scientific	AGS160-H2H
Protein-A gold 10 nm fiducials	Electron Microscopy Sciences	50-281-94

### Contact for reagent and resources sharing

Further information and requests for resources and reagents should be directed to and will be fulfilled by the Lead Contact, Helen Saibil (h.saibil@mail.cryst.bbk.ac.uk).

### Experimental model and subject details

#### Bacterial Culture

Recombinant HTT_ex1_-GFP was expressed as a His_6_-MBP-Asn_10_ fusion in *E. coli* Rosetta (DE3) competent cells (Merck Millipore; Billerica, MA). Cells were grown in LB media and expression was induced at an OD_600_ of 0.8 with 1 mM IPTG and grown overnight at 12**°**C. Cells were pelleted by centrifugation and resuspended in 150 mM NaCl, 50 mM Na_2_HPO_4_/NaH_2_PO_4_ (pH 7.4) and protease inhibitors (Roche). Cells were lysed by sonication using a VC 130 sonicator (Sonics and Materials; Newtown, CT) and the lysate was clarified by centrifugation at 4**°**C.

#### Yeast Culture

Yeast cells were grown using standard culturing techniques in YPD (1% yeast extract, 2% peptone, 2% glucose), or synthetic dropout media (0.7% yeast nitrogen base without amino acids, 2% glucose) for auxotrophic selection of plasmids. Excess adenine (100 mg/L) was included in all experiments. For induction of galactose-controlled expression, cells were grown in media containing raffinose as the sole carbon source for several doublings to an OD_600_ of 0.5-0.7 then washed and switched to media containing galactose as the sole carbon source to induce protein expression. Cells were grown at 30°C with shaking at 220 rpm.

#### Mammalian Cell Culture

HEK293 cells were maintained in DMEM (Thermo Fisher Scientific), supplemented with 10% FBS and maintained at 37°C, 5% CO_2_ atmosphere. Before live cell imaging, cells were switched to FluoroBrite DMEM (Thermo Fisher Scientific) containing doxycycline (150ng/mL).

### Method Details

#### Yeast Methods

Yeast strains for the analysis of HTT_ex1_ assemblies were derived from the 74-D694 background (MATα, ade1-14 ura3-52 leu2-3, 112 trp1-289 his3-Δ200; Chernoff et al., 1995). To generate the HTT_ex1_-eGFP expression plasmids, an N-terminal FLAG tag was added to existing sequences (Dehay and Bertolotti, 2006) by PCR amplification, and these were cloned into the 2μ expression plasmid p425GAL1 (Sikorski and Hieter, 1989) at SpeI-SalI sites. The integrity of all clones was confirmed by sequencing. As eGFP has a propensity to dimerize (Zacharias et al., 2002), we generated a 97QP construct fused to the monomeric fluorescent protein mEOS3.1 (Zhang et al., 2012), to confirm that we observed both types of assembly regardless of the fluorescent protein tag. The 97QP-mEOS3.1 construct was generated by splicing overhang extension (SOEing) PCR (Horton et al., 1989) of 97QP and mEOS3.1 fragments. To ensure that polyQ lengths were exactly matched between P-rich ± proteins, it was necessary to synthesize the sequences for 97Q-GFP and 43Q-GFP (GenScript). Transformation of cells was carried out using a standard lithium acetate method.

#### Prion Curing

Yeast cells were cured by passaging over 2% glucose YPD plates (see yeast methods) containing 5 mM Gd-HCl (Cox et al., 1988), 4 times, so that aggregation could be compared in isogenic [RNQ+]/[rnq-] strains. To confirm prion curing, we transformed the [RNQ+]/[rnq-] strains with a plasmid encoding Rnq1-GFP and induced its expression for 4 hr before checking cells by fluorescence microscopy. Rnq1-GFP formed aggregates in the [RNQ+] strain but not in the [rnq-] strain (Figure S3C), confirming that the [rnq-] strain had been cured of the prion form of Rnq1. [RNQ+] and [rnq-] strains both gave rise to the expected patterns of toxicity (Figure S5B; Meriin et al., 2002).

#### Yeast Toxicity Assay

Strains were grown to exponential phase in selective media with 2% glucose (see yeast methods), washed in sterile water, and normalized to an OD_600_ of 0.1. 4x 5-fold serial dilutions of cells were spotted onto selective medium agar plates containing galactose (induction) or glucose (repression). A multi-channel pipette was used to make sure that spots were aligned. Older plates were used to prevent the spots from running into one another when applying the dilutions (we used 5 μL per spot but this may require optimization depending on the concentration of cells and the composition of the plates). Plates were photographed 2-3 days later (Duennwald, 2012).

#### Yeast Expression Levels

After 4 or 24 hr induction, cell densities were normalized for all samples (OD_600_ measurement), pelleted by centrifugation (1800 g, 10 min) and re-suspended in cold lysis buffer (50 mM Tris HCl, pH 7.4, 150 mM NaCl, 0.5 mM DTT, 50 μg/ml heparin, 1:5000 antifoam A concentrate (Sigma-Aldrich; St. Louis, MO), 1 complete mini EDTA free protease inhibitor tablet per 50 mL (Roche; Basel, Switzerland)). Samples were lysed in lysis buffer by vortexing with 425-600 μm acid-washed glass beads (Sigma-Aldrich), for 6x cycles of vortexing (1 min) and cooling on ice (1 min). Lysates were cleared of cellular debris by centrifugation (800 g, 2 min), then 4x 5-fold serially diluted and applied to a 0.2 μm nitrocellulose membrane (Bio-Rad; Hercules, CA) using a Bio-Dot Apparatus (Bio-Rad). HTT_ex1_-GFP proteins were detected using a Living Colors A.v. Monoclonal Antibody JL-8 (Clontech Laboratories; Mountain View, CA), Goat anti-mouse IgG (H+L) secondary antibody, DyLight 488 (Thermo Fisher Scientific; Waltham, MA) and FLA-3000 fluorescent image analyzer (Fujifilm; Tokyo, Japan).

#### Quantitative Dot Blots

Yeast lysates were prepared as described above, serially diluted, and blotted onto nitrocellulose membranes, along with a series of ten 2-fold serial dilutions of purified 25QP-GFP at a known concentration (from a starting concentration of 2 μM). The resulting blots were probed and imaged as described above and analyzed in Fiji. First, the images were inverted and background subtracted using a rolling ball radius of 10. Equally sized circles, the sizes of dots, were centered on each dot and used to measure the raw integrated density of the fluorescence signal from each dot. The dots made by purified 25QP-GFP were used to generate calibration curves by plotting the raw integrated density against the known concentration of the protein (Figure 5B). The calibration curves were generated by fitting the data to a hyperbolic curve,

$$Raw\;integrated\;density=\frac{a\;\times \;\left[protein\right]}{b+\left[protein\right]}$$

with a and b being free parameters (Janes, 2015). The concentrations of HTT_ex1_ proteins in the lysates were determined using the calibration curve generated from the same blot. The intracellular concentrations of HTT_ex1_ proteins were then estimated based on the number of cells contributing to each dot.

#### Mammalian Cell Methods

DNA sequences for the HTT_ex1_-GFP constructs were taken from the yeast expression plasmids and inserted into the pcDNA5-FRT-TO (Thermo Fisher Scientific) mammalian expression vector allowing doxycycline-inducible expression of the construct. At 80%–90% confluency, cells were transfected with expression plasmids using Lipofectamine 2000 (Thermo Fisher Scientific) according to the manufacturer’s protocol, and HTT_ex1_-GFP expression was induced using doxycycline (150 ng/mL). The Flp-In HEK293 HTT_ex1_-GFP (25 and 97QP) isogenic stable cell lines were generated using Flp-In System (Invitrogen) following the manufacturer’s protocol. Briefly, pcDNA5-FRT-TO expressing HTT_ex1_-GFP constructs were co-transfected with pOG44 plasmid (Invitrogen), which constitutively expresses the Flp recombinase. Stable Flp-In expression cell lines were selected for hygromycin resistance. HTT_ex1_-GFP was induced using doxycycline (150ng/mL).

#### Arsenite-induced Stress Granule Formation and Immunofluorescence

Cells were plated on glass coverslips coated with poly-D-Lysine. 24h after induction of 97QP-GFP, stress granule assembly was stimulated by addition of 0.5 mM of sodium arsenite for 45 min at 37°C, 5% CO_2_. After stress, cells were washed three times with PBS and fixed with 4% PFA in PBS for 10 min at room temperature, permeabilized with 0.5% Triton in PBS for 5 min at room temperature, washed with PBS and incubated with blocking buffer (4% BSA in PBS) for 1h at room temperature. Primary antibodies were diluted in blocking buffer and incubated for 1 h at room temperature. To monitor stress granule formation, goat anti-TIA-1 (Santa-Cruz, Dallas, TX; SC-1751; 1/100) and mouse anti-G3BP (BD Biosciences, Franklin Lakes, NJ; #611126; 1/1000) were used. After washing with 0.1% Tween in PBS, cells were incubated with IgG (H+L) secondary antibodies donkey anti-goat Alexa 647 or donkey anti-mouse Cy3 (Thermo Fisher Scientific, 1/500) for 45 min. Coverslips were mounted in Prolong Gold Antifade mounting medium (Thermo Fisher Scientific).

#### Time-lapse Experiments

HEK293 cells were initially plated and transfected in 35 mm glass-bottomed dishes No. 1.5 (MatTek; Ashland, MA) coated with poly-D-lysine. To begin induction, the medium was switched to FluoroBrite DMEM (Thermo Fisher Scientific), supplemented with 10% FCS containing doxycycline (150 ng/mL). Cells were allowed to incubate for 2 hr then transferred to an incubator microscope (described below). Cells were maintained at 37**°**C, 5% CO_2_ and imaged for 24-48 hr. Typically, we imaged around 10 positions of a dish with a frame rate of ∼3 mins.

#### Incubator Microscope

A custom-built automated epifluorescence microscope was built inside a standard CO_2_ incubator (Thermo Fisher Scientific; Heraeus BL20) that maintained the temperature at 37°C and in a 5% CO_2_ atmosphere. The microscope comprised a high performance motorized stage (Prior Scientific, Cambridge, UK; Proscan III, H117E2IX), with a motorised focus controller (Prior; FB201 and PS3H122R) and a 9.1MP CCD camera (FLIR, Wilsonville, OR; Point Grey GS3-U3-91S6M). Brightfield illumination was provided using a green LED (Thorlabs, Newton, NJ; M520L3, 520 nm). Fluorescence illumination in two channels, GFP and mCherry/RFP, was via a blue (Thorlabs; M470L3, 470 nm) or yellow (Thorlabs; M565L3, 565 nm) LED respectively. These were combined using a dichroic beamsplitter (Semrock, Rochester, NY), and focused onto the back focal plane of a 20x air objective (Olympus 20x, 0.4NA) in an epifluorescence configuration. The camera and the LEDs were synchronized using TTL pulses from an external D/A converter (Data Translation, Marlborough, MA; DT9834). A custom built humidified chamber maintained the humidity around the sample and was fitted with a thermocouple and humidity sensor to continuously monitor the environment.

#### Single Cell Tracking

Computational cell tracking was performed as previously described (Bove et al., 2017), using the time-lapse movies that were collected on the incubator microscope described above. Briefly, we trained a deep convolutional neural network (CNN) to segment cells from movie frames based on a training dataset of ∼500 manually segmented cells. The only input to the network was the bright-field transmission channel. To increase the number of training examples, we augmented the examples by introducing random transformations, rotations, and flips. Training was performed using a momentum optimizer with an exponentially decaying learning rate until convergence. We used dropout (50% while training) to prevent over-fitting. The CNN was implemented using Tensorflow (https://www.tensorflow.org) (Abadi et al., 2016). Next, the centroids of each segmented cell were tracked using a Bayesian tracking method (https://github.com/quantumjot/BayesianTracker). The tracking procedure resulted in trajectories of single cells over time. Well-tracked cells were then selected and a custom Python (https://www.python.org) script was used to generate the time-resolved histograms of fluorescence intensity, using the segmentation output as a mask. These were further analyzed and plotted using R (https://www.r-project.org).

#### Hexanediol Experiments

Yeast cells were grown in the appropriate selection media and expression of HTT_ex1_ constructs was induced with galactose (see yeast methods) until assemblies formed. Yeast cells were adhered to concanavalin A (Sigma) coated chamber slides (μ-Slide VI^0.4^; Ibidi, Germany) and unbound cells were removed by washing. Slides were mounted on a custom-built TIRF microscope (see Total Internal Reflection Fluorescence (TIRF) Microscopy) set up in a wide-field fluorescence configuration, with the temperature maintained at 30°C. Time-lapse movies with a frame rate of 5 s were acquired as solutions of either 10% 1,6-hexanediol (Sigma) + 10 μg/μL digitonin (Sigma), or digitonin alone, in yeast growth medium, were injected into the chambers. Hexanediol removal was done by injecting excess fresh yeast growth medium back into the chambers. Movies were analyzed in Fiji (Schindelin et al., 2012). Bleach correction was carried out with the Bleach Correction plugin (https://imagej.net/Bleach_Correction), using the simple ratio method and making use of cells that did not contain assemblies. Kymographs were generated using the Fiji “reslice” command and the change in fluorescence was quantified over time and plotted using R.

#### FRAP Methods

FRAP was performed using a Leica TCS SP5 microscope equipped with an HCX Plan-Apochromat lambda blue 63x oil-immersion objective (NA 1.4). Intracellular assemblies were bleached in a circular 0.28 μ^2^ region of interest using a 0.2 s pulse of the 488 nm laser line at full power. Recovery was monitored every 0.116 s for 400 frames. The recovery curves were analyzed using the jython script, http://fiji.sc/Analyze_FRAP_movies_with_a_Jython_script, in Fiji. Plotting and error calculations were done in R, and curve fitting was carried out in MATLAB. For yeast FRAP, analysis was performed using the FRAP Profiler plugin in Fiji (http://worms.zoology.wisc.edu/research/4d/4d.html#frap), which normalizes for overall bleaching and therefore avoids inaccurate estimation of mobile/immobile fractions in small cells, where bleaching can result in a significant reduction in total cellular fluorescence. For *in vitro* half-bleaching (Brangwynne et al., 2009) of 25QP-GFP droplets, an area corresponding to half of a droplet was bleached and recovery was monitored every 0.2 s for 15 s. See also Tables S1 and S2.

#### Intensity and Circularity Ratios

Intensity ratios for individual cells were calculated in Fiji from confocal slices through assemblies, by measuring the mean fluorescence intensity in circular areas of 1 μ^2^ in the assembly and in the cytosol, and dividing the two values:

$$IR=\;\frac{I_{assembly}}{I_{cytosol}}$$

Further statistical analysis was performed in R.

The circularity ratio (CR) of an object is a measurement of how circular it appears: the ratio of the area of the object to the area of a perfect circle with the same perimeter as the object, given by

$$CR=4\pi \left(\frac{Area}{Perimeter^{2}}\right)$$

where a perfectly circular object has a CR of 1 and decreasing circularity tends toward a CR of 0. CRs of assemblies were measured from images of confocal slices through assemblies which were cropped and auto-thresholded in Fiji using the Huang method (Huang and Wang, 1995). The area and perimeter of the thresholded assemblies was measured and imported into R where the CRs were calculated.

#### EM Sample Preparation

Yeast cells were pelleted by centrifugation (3000 g, 10 min) and re-suspended in growth medium to produce a wet slurry. High-pressure freezing and freeze-substitution was performed as previously described (O’Driscoll et al., 2015).

Mammalian cells were gently re-suspended then pelleted by centrifugation (300 g, 3 min). 3-5 μL aliquots of the loose pellet were immediately transferred to Leica Cu-Au specimen carriers (Type A, 0.2 mm cavity; Type B, flat side) and high-pressure frozen with an EM HPM100 (Leica). Subsequent freeze-substitution, ultramicrotomy, and post-staining procedures were carried out as for yeast.

#### Correlative Light and Electron Microscopy

HM20 resin-embedded cell sections were mounted on glow-discharged S160-H2H carbon-coated copper finder grids (Agar Scientific; Essex, UK). The grids were wet-mounted as previously described (Kukulski et al., 2011) and mapped by light microscopy using the fluorescence and DIC channels of the axioscope A1 microscope described in the ‘sequence effects experiment’ section. After mapping, grids were stained (see EM sample preparation) and imaged by EM. Correlation between fluorescence and low-magnification EM images was performed in Fiji using an affine transformation between corners of the grid square (TurboReg plugin: http://bigwww.epfl.ch/thevenaz/turboreg/; Thévenaz et al., 1998). Fluorescence images were then transformed onto EM images and a series of transformations was calculated through an EM magnification series up to the magnification of the tilt series, using internal sample fiducial markers.

#### Electron Tomography Data Collection and Processing

Grids were mounted on a Model 2040 dual-axis tomography holder (Fischione Instruments; Export, PA) and imaged using a Tecnai T12 microscope (FEI; Hillsboro, OR) operated at full voltage and equipped with a Ultrascan US4000 4k CCD camera (Gatan; Abingdon, UK). Acquisition of dual-axis tilt series was controlled using SerialEM software (Mastronarde, 2005). Tilt-series were typically collected over a tilt range of ±60° with 2° increments, at calibrated unbinned pixel sizes of 0.456 nm (26kx magnification) or 0.514 nm (21kx magnification), with a defocus of 0.8 μm. Tomograms were reconstructed from tilt series by weighted back-projection using the IMOD package, version 4.9 (Kremer et al., 1996).

#### Sequence Effects Experiment

To study the effect of HTT_ex1_ sequence on assembly formation, [rnq-] yeast cells transformed with 25Q-GFP, 25QP-GFP, 43Q-GFP, 43QP-GFP, 97Q-GFP or 97QP-GFP plasmids were grown in parallel to exponential phase, then induced with galactose (see yeast methods). Thereafter, cells were examined by wet-mount on poly-lysine slides (VWR International LLC) at 4, 8, 12 and 24 hr post-induction, using an Axioscope A1 epifluorescence microscope (Carl Zeiss; Oberkochen, Germany), equipped with an X-Cite Series 120Q lamp and an Orca R2 CCD camera (Hamamatsu Photonics; Hamamatsu City, Japan), with a 63x Plan-Apochromat oil-immersion objective (NA 1.4). Fluorescence images were taken using the 470/40 nm and 525/50 nm excitation and emission filters. DIC images were also taken. Cells containing a) LAs b) SAs or c) diffuse fluorescence were manually counted for each time point and construct, and the data were plotted using R.

#### Protein Purification

The filtered lysate (see Experimental Model and Subject Details: Bacterial Culture) was loaded onto a 5 mL Ni HisTrap column (GE Healthcare; Chicago, IL). The protein was eluted with a linear imidazole gradient (5 – 250 mM imidazole), concentrated and loaded onto a HiLoad 16/60 Superdex 200 size exclusion chromatography column (GE Healthcare) that had been equilibrated with 150 mM NaCl, 50 mM Na_2_HPO_4_/NaH_2_PO_4_ (pH 7.4). Fractions containing 25QP-GFP were pooled and dialysed overnight with TEV protease to remove the tag. The tag was separated from 25QP-GFP by Ni affinity chromatography with a 5 mL Ni HisTrap column (GE Healthcare). Pure, concentrated 25QP-GFP was flash frozen in liquid nitrogen and stored at −80**°**C until use. 43QP-GFP was purified in the same way as 25QP-GFP except that an MBPTrap (GE Healthcare) chromatography step was added at the end to ensure complete removal of the cleaved tag.

#### *In vitro* Experiments

Phase separation of 25QP-GFP was induced by molecular crowding (Patel et al., 2015), by mixing the purified protein (150 mM NaCl, 50 mM Na_2_HPO_4_/NaH_2_PO_4_, pH 7.4) with 20% dextran (64 – 76 kDa; Sigma), 150 mM NaCl, 50 mM Na_2_HPO_4_/NaH_2_PO_4_, pH 7.4 in a 1:1 volume ratio in a PCR tube at room temperature. For imaging, the mixture was allowed to equilibrate for 3 mins, then transferred to glass slides that had been treated with 30 mg/ml BSA (Sigma) for 30 mins.

#### Phase Diagrams

20% dextran buffers, with different salt or hexanediol concentrations were made up and mixed with different dilutions of 25QP-GFP in a 1:1 volume ratio, to give the desired protein, salt, and hexanediol concentrations. Droplet formation was assessed by fluorescence and DIC microscopy (see *in vitro* experiments) on the A1 axioscope microscope described above (sequence effects experiment). Each condition was tested at least twice.

#### Total Internal Reflection Fluorescence (TIRF) Microscopy

TIRF microscopy was performed using a custom built microscope, based on an Olympus IX81 frame (Olympus, Japan) with a high NA oil objective (Olympus 100x UPON TIRF 1.49 N.A. oil). An air-cooled EMCCD camera (Andor, Belfast, UK; iXon Ultra DU-897U-CS0-#BV) or sCMOS camera (Hamamatsu; Orca Flash4.0V2) was coupled to the camera port of the microscope via a magnifying relay to achieve an effective pixel size of ∼100 nm. Appropriate bandpass filters were placed in front of the camera to filter the fluorescence emission. A fiber-coupled CW laser (Toptica, Munich, Germany; iChrome HP, 488, 561, and 640 nm) was expanded using a collimating lens and relayed via the camera port of the microscope via a fast steering mirror (Newport, Irvine, CA; FSM-100) positioned at a conjugate image plane. The fast-steering mirror is steered at the critical TIRF angle and around the back focal plane of the objective at high speed to yield improved illumination homogeneity. The camera and FSM were synchronized using a TTL pulse and analog signal from an external D/A converter (Data Translation; DT9834). A quarter-wave plate was used to circularly polarize the beam before it was injected into the microscope. The beam was directed to the objective lens via a multi-edge dichroic filter (Semrock; Di01-R405/488/561/635-25x36). An objective heater and heated sample chamber (Okolab; Pozzuoli, Italy) were used to maintain sample temperature.

#### Liquid to Solid Conversion Experiment

After droplet formation, an aliquot of the reaction mixture (200 μM 25QP-GFP in 10% dextran, 150 mM NaCl, 50 mM Na_2_HPO_4_/NaH_2_PO_4_, pH 7.4) was added to a glass-bottomed dish (35mm dish, 14mm glass diameter; MatTek) that had been treated with 30 mg/mL BSA (Sigma) for 30 mins. The dish was sealed to prevent evaporation and the droplets were imaged by time-lapse fluorescence microscopy using the custom-built TIRF microscope described above (Total Internal Reflection Fluorescence (TIRF) Microscopy), set up in a configuration with the fast steering mirror angled to improve signal-to-noise ratio. Images were collected every 10 s for 1 – 2 hr until the droplets had converted. The laser was operated at 0.1% power (∼0.1mW) and was further reduced in power by placing a half-wave plate and polarizing beam splitter (Thorlabs) in the beam path. Experiments were carried out at room temperature (21°C).

The circularity of the droplets over time was measured by applying automatic local thresholding to each frame using the Otsu method (Otsu, 1979) in Fiji to create a binary image of the droplets, then measuring the areas and perimeters. CRs were calculated as described (Intensity and circularity ratios) in R and the mean CR in the field of view was plotted over time.

#### Droplet Dissolution using Hexanediol

Droplets were prepared and imaged by time-lapse fluorescence microscopy as described above (see liquid to solid conversion experiment). Droplet conversion was allowed to progress for 20 mins then an aliquot of dissolution buffer (10% hexanediol, 150 mM NaCl, 50 mM Na_2_HPO_4_/NaH_2_PO_4_, pH 7.4) was added to the conversion reaction in a 1:2 ratio of dissolution buffer: reaction, by volume.

Selected frames, before and after droplet dissolution, were median filter background subtracted in Fiji for display purposes. To quantify the positions of the solid component in droplets that had not yet grown spikes, linescans of individual droplets before and after hexanediol addition were used to calculate the radial shift in maximum fluorescence intensity i.e., the location of the solid component relative to the center of the droplet.

### Quantification and Statistical Analysis

Statistical significance and the methods used to determine it are described in the figure legends of the relevant experiments. No methods were used to determine sample size, or strategies for randomization. No data were excluded.

## Supplemental Information

Document S1. Figures S1–S7 and Tables S1 and S2

Video S1. Time-Lapse Microscopy of 97QP-GFP Aggregation, Related to Figure 1 Time-lapse fluorescence microscopy shows the formation of a dim assembly that remains intact during division of a HEK293 cell. The dim assembly then converts to a bright assembly, which rapidly sequesters the cytoplasmic fluorescence in the affected cell.

Video S2. 3D Projections of Bright and Dim Assemblies, Related to Figure S1G 3D projections were generated from confocal stacks through the assemblies. Scale bar, 4 μm.

Video S3. Tomogram of a 97QP-GFP Bright Assembly, Related to Figure 1 3D volume through a bright assembly in a HEK293 cell. The assembly is made up of a dense meshwork of fibers. The assembly is not membrane bound and it generally excludes ribosomes. Double membrane (right) is the nuclear envelope.

Video S4. Dim Assemblies Show Liquid-like Behavior, Related to Figure 3D Hexanediol was added to a yeast cell, causing the dim assemblies to dissolve. When hexanediol was washed away, the assemblies rapidly reformed. Time-lapse fluorescence microscopy (5 s frame rate) was used to record the movie.

Video S5. Fusion of Droplets *In Vitro*, Related to Figure 6 Recombinant 25QP-GFP was exposed to 10% dextran to induce droplet formation. TIRF microscopy shows droplets fusing and relaxing back into a spherical shape.

Video S6. Liquid- to Solid-like Conversion of Droplets, Related to Figure 7 Time-lapse TIRF microscopy shows the conversion of 25QP-GFP droplets to solid-like structures. Note the growth of spikes from the edges of the droplets and the depletion of the liquid-like component at the centers of droplets.

Video S7. Droplet Dissolution by Hexanediol, Related to Figure 7 Time-lapse TIRF microscopy of hexanediol addition to 25QP-GFP droplets. The hexanediol moves across the field of view, dissolving the liquid-like component of the droplets and leaving behind elongated structures that are likely early solid-like features.

Document S2. Article plus Supplemental Information
