# Supplementary material for: A Liquid to Solid Phase Transition Underlying Pathological Huntingtin Exon1 Aggregation
Source: Mol Cell. 2018 May 17;70(4):588–601.e6. doi: 10.1016/j.molcel.2018.04.007 (PMC5971205; doi:10.1016/j.molcel.2018.04.007)
Supplement: Document S1. Figures S1–S7 and Tables S1 and S2 [file mmc1.pdf]

Molecular Cell, Volume *70*

Supplemental Information

A Liquid to Solid Phase Transition Underlying Pathological Huntingtin Exon1 Aggregation

Thomas R. Peskett, Frédérique Rau, Jonathan O'Driscoll, Rickie Patani, Alan R. Lowe, and Helen R. Saibil

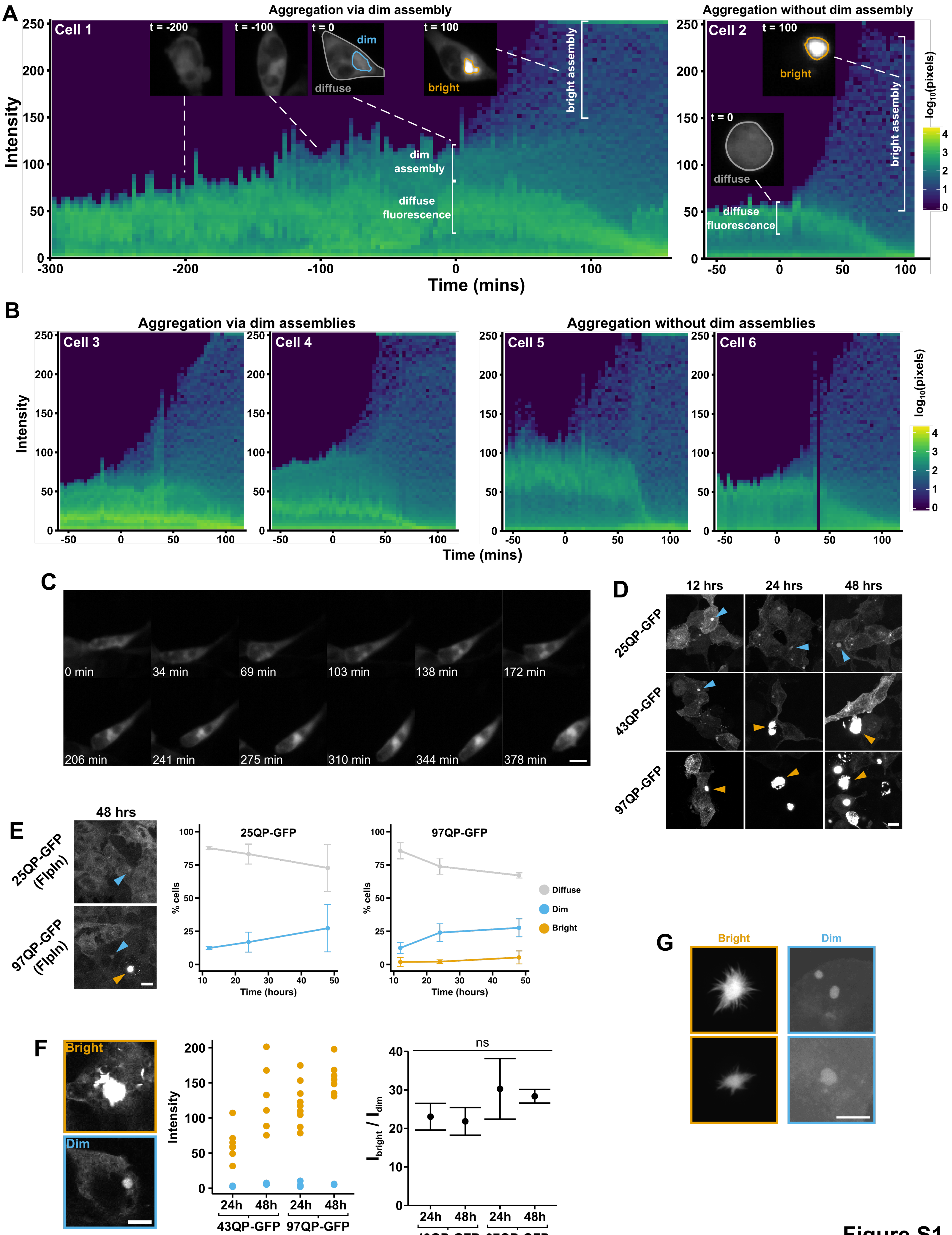
A
Aggregation via dim assembly
Aggregation without dim assembly
Cell 1
t = -200
t = -100
t = 0
dim
diffuse
t = 100
bright
bright assembly
dim assembly
diffuse fluorescence
Cell 2
t = 100
bright
t = 0
diffuse
bright assembly
diffuse fluorescence
Intensity
Time (mins)
log10(pixels)
B
Aggregation via dim assemblies
Aggregation without dim assemblies
Cell 3
Cell 4
Cell 5
Cell 6
Intensity
Time (mins)
log10(pixels)
C
0 min
34 min
69 min
103 min
138 min
172 min
206 min
241 min
275 min
310 min
344 min
378 min
D
12 hrs
24 hrs
48 hrs
25QP-GFP
43QP-GFP
97QP-GFP
E
48 hrs
25QP-GFP (FlpIn)
97QP-GFP (FlpIn)
25QP-GFP
97QP-GFP
% cells
Time (hours)
Diffuse
Dim
Bright
F
Bright
Dim
Intensity
24h
48h
24h
48h
43QP-GFP
97QP-GFP
ns
I_bright / I_dim
G
Bright
Dim

Figure S1

Figure S1, related to Figure 1.

(A) Quantification of aggregation trajectories in single cells. Cells were computationally tracked and their fluorescence intensity distributions plotted as time-resolved histograms. t = 0 is defined as the point at which a bright assembly begins to form. Formation of bright assemblies corresponds with a rapid increase (~1hr) in intensities of a small subset of pixels. As bright assemblies form, highly populated intensities (diffuse fluorescence) reduce to zero in agreement with sequestration of cytoplasmic HTT_{ex1} into the forming assembly. Dim assemblies result in a subset of pixels occupying slightly higher intensities than the diffuse background fluorescence. In contrast to bright assemblies, they do not cause a loss of the cytoplasmic fluorescence. (B) Additional examples of single-cell aggregation trajectories. (C) Time-lapse fluorescence microscopy of merging 25QP-GFP assemblies. Scale bar, 10 µm. (D) 25QP-GFP forms dim assemblies but not bright assemblies. Cells were fixed at 12, 24 and 48 hrs after inducing expression of the constructs indicated. Bright and dim assemblies are highlighted with orange and blue arrows, respectively. Scale bar, 10 µm. (E) Quantification of fluorescent features in HEK293 Flp-In stable cell lines expressing 25QP-GFP or 97QP-GFP. Cells were fixed and imaged at 12, 24 and 48 hrs post-induction. (F) Quantification of mean intensities of bright and dim assemblies in 43QP-GFP and 97QP-GFP transfected HEK293 cells determined from confocal slices at 24 hr and 48 hr time points post-induction. The mean intensities of bright assemblies (orange) and dim assemblies (blue) cluster and do not overlap ($p < 0.0015$, Welch's two-sample t-test). Ratios of bright and dim intensities do not vary significantly ($p > 0.24$) between experiments. Assemblies depicted were acquired with the same settings but were not used for quantification due to pixel saturation in the bright example. Quantification was done using images acquired with a lower laser power. Scale bar, 5 µm. (G) Maximum intensity projections of confocal stacks through bright and dim assemblies. Compare the spiky, irregular edges of the bright assemblies to the smooth edges of the dim assemblies. The images of the dim assemblies have been linearly scaled for clarity. Related to Movie 2. Scale bar, 5 µm.

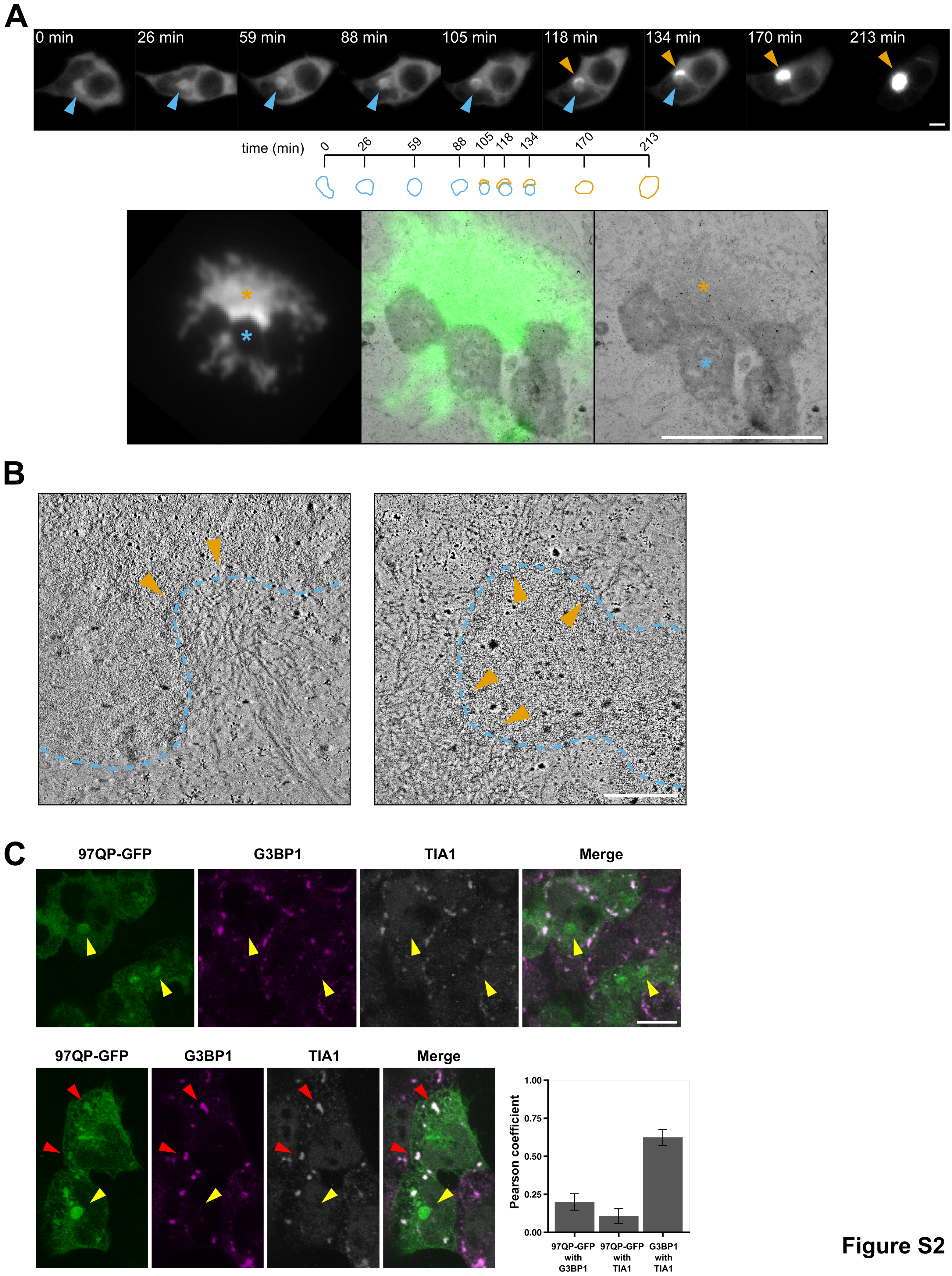
A
0 min
26 min
59 min
88 min
105 min
118 min
134 min
170 min
213 min
time (min)
0
26
59
88
105
118
134
170
213
B
C
97QP-GFP
G3BP1
TIA1
Merge
97QP-GFP
G3BP1
TIA1
Merge
Pearson coefficient
1.00
0.75
0.50
0.25
0.00
97QP-GFP with G3BP1
97QP-GFP with TIA1
G3BP1 with TIA1

Figure S2

Figure S2, related to Figure 1.

(A) Images from a time-lapse fluorescence microscopy experiment (top) showing a 43QP-GFP dim assembly (blue arrows) converting to a bright assembly (orange arrows). Note the appearance of the bright assembly at the edge of the dim assembly, and sequestration of cytoplasmic fluorescence by the bright assembly. The timeline (below) shows the relative positions of the frames in time, with manually traced outlines of the dim (blue) and bright (orange) assemblies. Large panels (bottom) show, from left to right: fluorescence of a bright assembly in a 120 nm section through a cell on an electron microscopy grid; an overlay of the fluorescence with the electron micrograph of the same assembly; the electron micrograph without the fluorescence overlay. Probable dim and bright assemblies are indicated with blue and orange asterisks, respectively. Scale bars, 5 µm. (B) Electron tomography of dim assemblies (blue dashes). Images show individual slices through tomograms. Fibres can be seen making direct contact with the edges of the dim assemblies (orange arrows). Related to Figure 1I. Scale bar, 500 nm. (C) Examples of coexisting 97QP-GFP dim assemblies and arsenite-induced stress granules (immunolabelled for G3BP1 and TIA1). Yellow arrows indicate dim assemblies that do not co-localise with stress granules. Red arrows indicate dim assemblies that do co-localise with stress granules. Images are of maximum intensity projections of confocal stacks. Scale bar = 10 µm. Graph shows Pearson coefficients for co-localisation of the proteins indicated. Pearson coefficients were calculated using the unmodified confocal stacks.

Figure S3

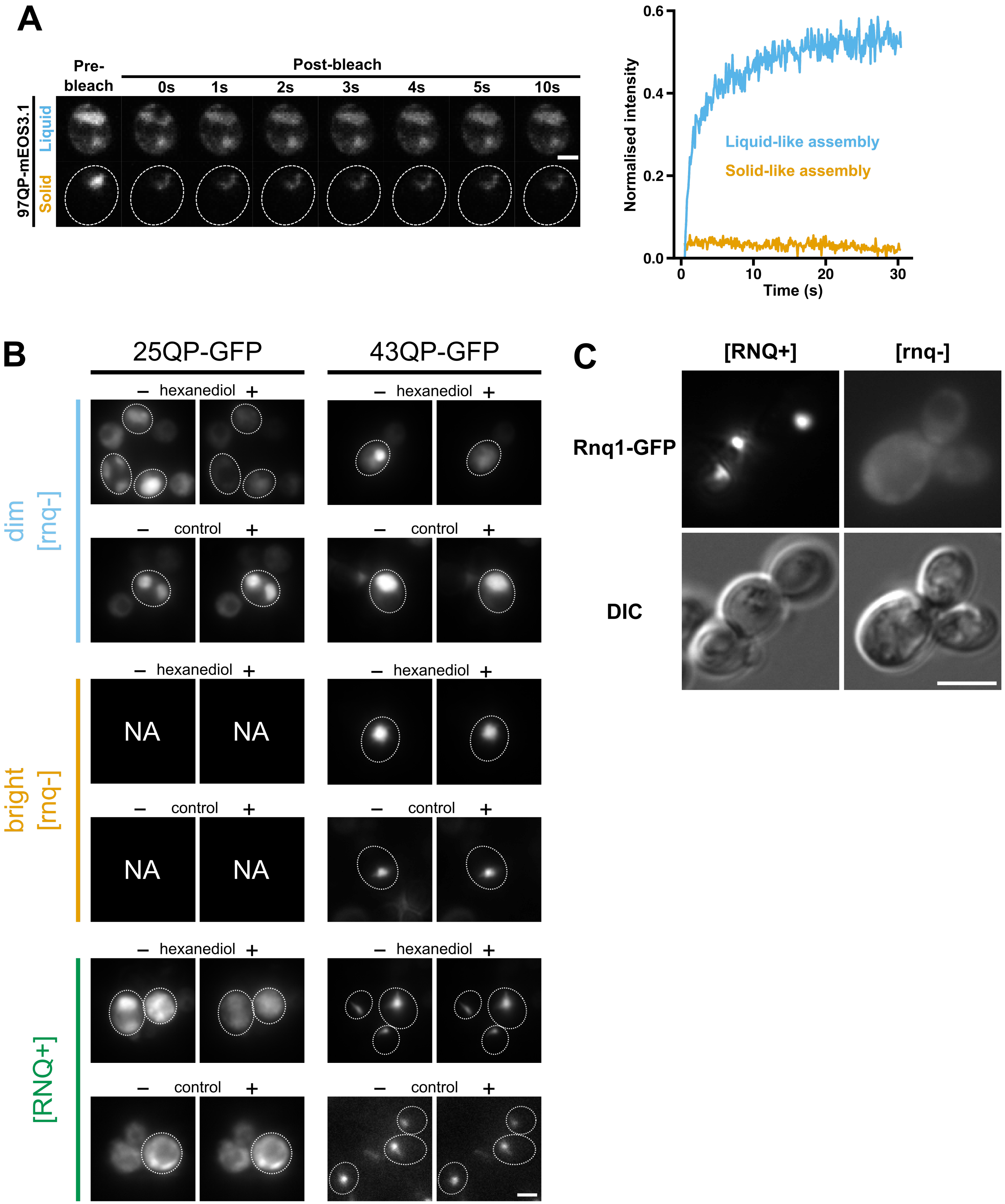

A
Pre-bleach
Post-bleach
0s
1s
2s
3s
4s
5s
10s
97QP-mEOS3.1
Liquid
Solid
0.6
0.4
0.2
0.0
Normalised intensity
0
10
20
30
Time (s)
Liquid-like assembly
Solid-like assembly
B
25QP-GFP
43QP-GFP
− hexanediol +
− control +
dim [rnq-]
bright [rnq-]
[RNQ+]
NA
C
[RNQ+]
[rnq-]
Rnq1-GFP
DIC

Figure S3, related to Figures 2 and 3.

(A) Yeast cells in the [rnq-] background expressing 97QP-mEOS3.1 were induced for 4 hrs and the resulting assemblies were probed by FRAP. Example FRAP curves show the two distinct assembly types. Note that the bleached area of the liquid-like assembly recovers rapidly and that the mobile fraction is under-represented due to the significant bleaching of the total cellular fluorescence. See also **Table S1**. (B) Fluorescence microscopy of 25QP-GFP and 43QP-GFP HTT_{ex1} assemblies before (-) and after (+) addition of hexanediol. Controls were performed by permeabilising cells in the absence of hexanediol. White dotted outlines indicate positions of cells. Colours indicate the Rnq1 prion status and, in the case of [rnq-] cells, the type of assembly. Note that 25QP-GFP did not form SAs (panels labelled 'NA'). Scale bar, 3 µm. (C) [RNQ+] yeast cells were cured of the prion form of Rnq1 by four passages over 5 mM Gd-HCl plates (Cox et al., 1988; see materials and methods), to generate a [rnq-] strain. Prion curing was confirmed by expressing Rnq1-GFP in both strains and visualising the distribution of Rnq1-GFP using widefield fluorescence microscopy: in the [RNQ+] strain, Rnq1-GFP formed puncta whereas in the [rnq-] strain it had a diffuse distribution. Scale bar, 5 µm.

Figure S4

25QP-GFP [rnq-]

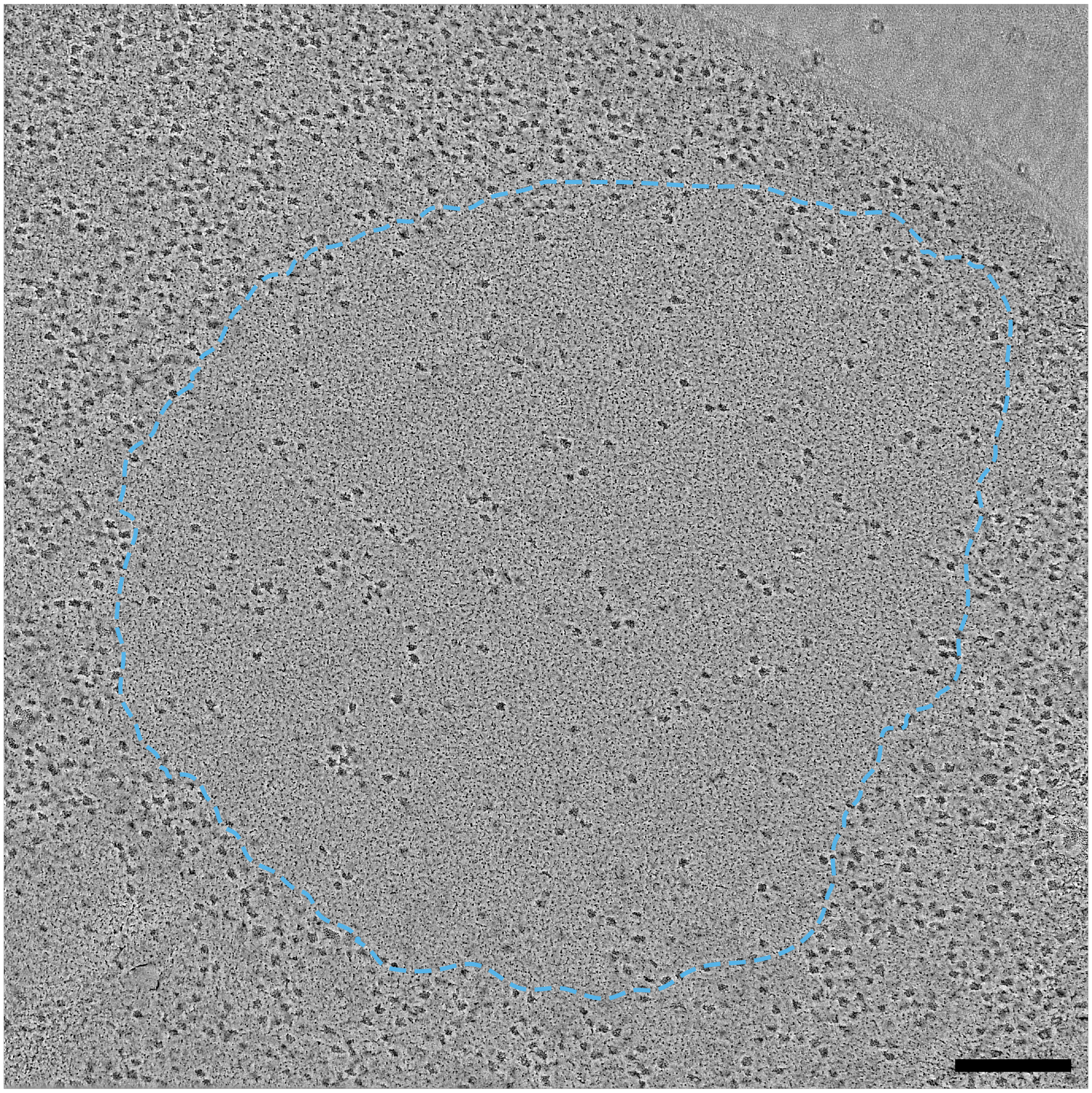

Figure S4, related to Figure 4.

Slice through a tomogram of a 25QP-GFP liquid-like assembly (outlined in blue) formed in a yeast cell. Scale bar, 200 nm.

Figure S5

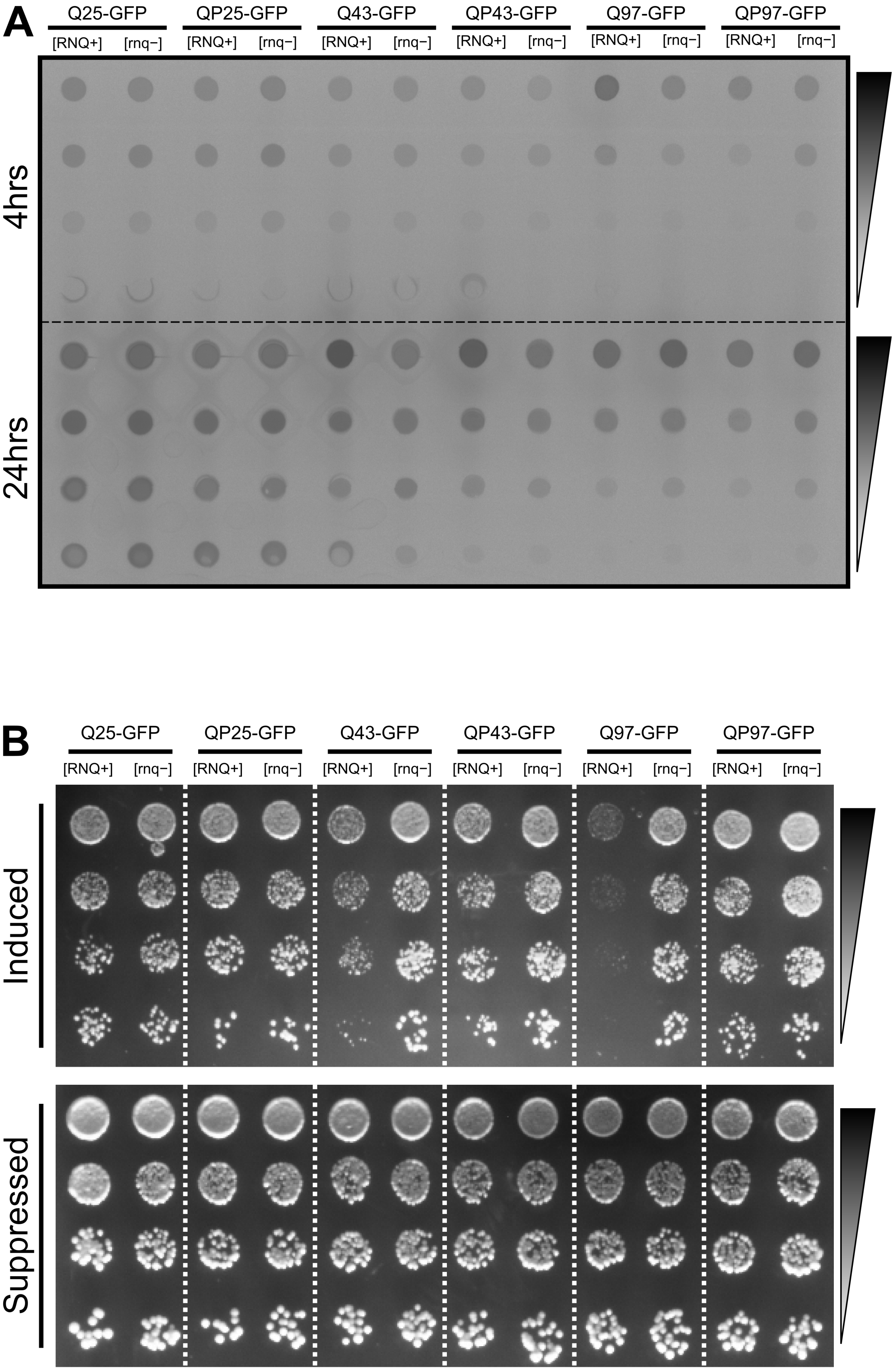
A
Q25-GFP
QP25-GFP
Q43-GFP
QP43-GFP
Q97-GFP
QP97-GFP
[RNQ+]
[rnq−]
4hrs
24hrs
B
Q25-GFP
QP25-GFP
Q43-GFP
QP43-GFP
Q97-GFP
QP97-GFP
[RNQ+]
[rnq−]
Induced
Suppressed

Figure S5, related to Figure 5 and STAR Methods.

(A) Dot blots of total HTT_{ex1} extracted from yeast cells after 4 and 24 hrs induction. Each sample is blotted as 4x 5-fold serial dilutions. (B) Yeast growth-based toxicity assay. Equal volumes of the indicated samples were spotted onto synthetic dropout agar plates lacking leucine, as 4x 5-fold serial dilutions. HTT_{ex1} proteins were induced or supressed by using galactose or glucose, respectively, as the sole carbon source. As previously shown (Dehay and Bertolotti, 2006; Duennwald et al., 2006b), the proline-rich region suppresses toxicity; HTT_{ex1} is only toxic in the [RNQ+] background (Meriin et al., 2002); and polyQ length correlates with toxicity in the [RNQ+] strains lacking the proline-rich region (Dehay and Bertolotti, 2006; Duennwald et al., 2006b).

Figure S6

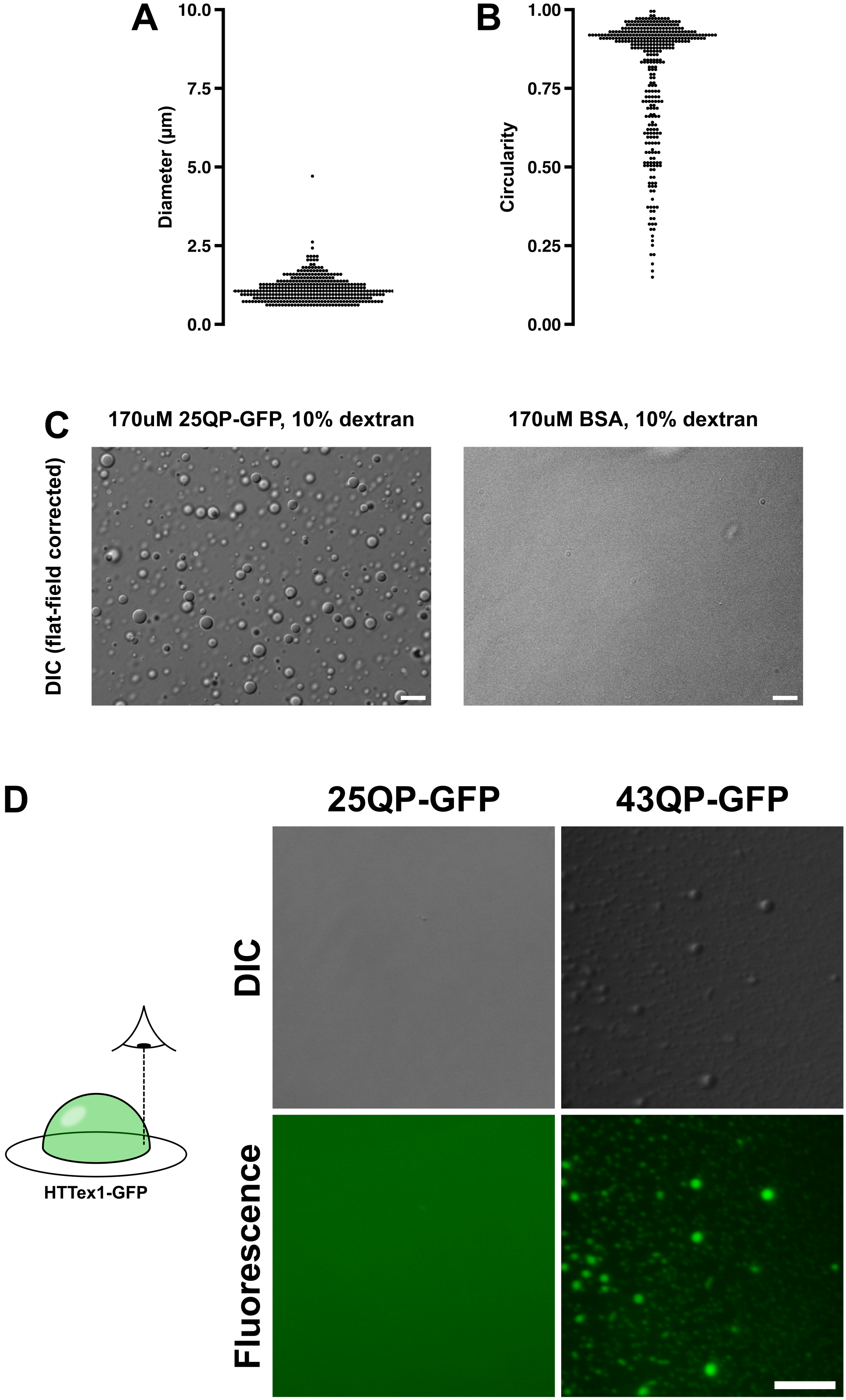
A
10.0
7.5
5.0
2.5
0.0
Diameter (μm)
B
1.00
0.75
0.50
0.25
0.00
Circularity
C
170uM 25QP-GFP, 10% dextran
170uM BSA, 10% dextran
DIC (flat-field corrected)
D
25QP-GFP
43QP-GFP
DIC
Fluorescence
HTTex1-GFP

Figure S6, related to Figure 6.

(A) Diameters of droplets, where each dot represents a single droplet. (B) Circularity ratios of droplets, where each dot represents a single droplet. (C) BSA does not form droplets under crowding conditions that induce 25QP-GFP to form droplets. Images were flat-field corrected against a background image acquired in the presence of the buffer. Scale bar, 10 µm. (D) Drops of purified 25QP-GFP and 43QP-GFP (60 µM) in the absence of crowding agent were placed on glass slides and imaged immediately at the sample edges. The 43QP-GFP sample formed micron sized assemblies with a variety of morphologies including rounded droplets and elongated spikes.

Figure S7

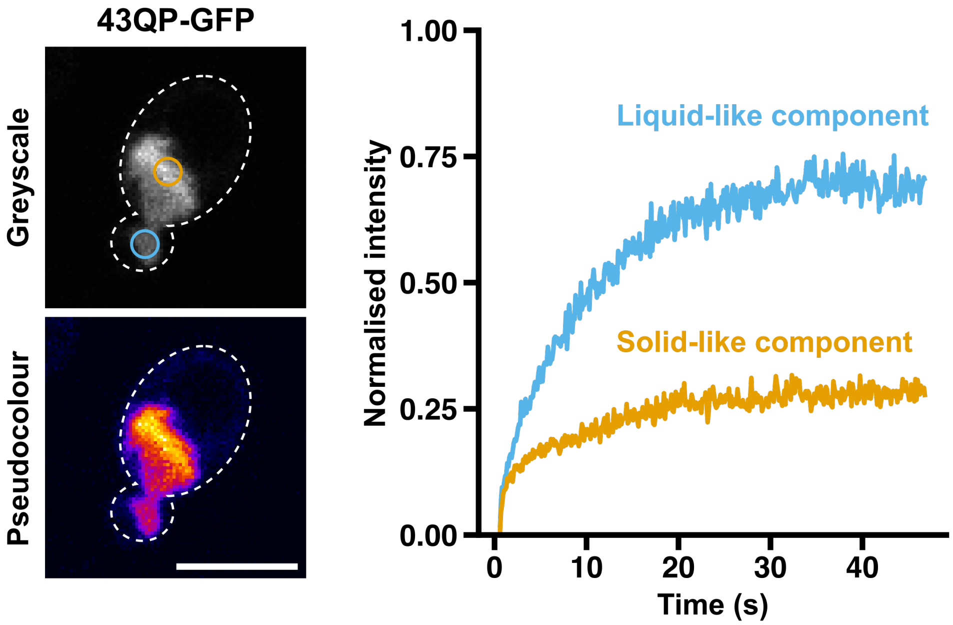

43QP-GFP
Greyscale
Pseudocolour
Liquid-like component
Solid-like component
Normalised intensity
1.00
0.75
0.50
0.25
0.00
0
10
20
30
40
Time (s)

Figure S7, related to Figure 7.

The images show a 43QP-GFP LA in a yeast cell (white dotted outline), undergoing a conversion to an SA. FRAP was performed on the regions indicated by the blue and orange circles in the greyscale image. The graph (right) shows the resulting recovery curves, whose colours correspond to the bleach region circles. The pseudocolour image highlights the different fluorescence intensities in the liquid- and solid-like regions of the converting assembly. Scale bar, 5 µm.

Table S1 FRAP recovery curves modelled using first and second order exponential functions. Related to Figures 1G, 2B, 7C and S3A.

Sample	Equation	A_1	A_2	τ_1 (s)	τ_2 (s)
43QP-GFP mammalian dim	1st order exponential	0.83	N/A	0.79	N/A
43QP-GFP mammalian bright	1st order exponential	0.10	N/A	0.96	N/A
43QP-GFP yeast dim	1st order exponential	0.86	N/A	2.41	N/A
43QP-GFP yeast bright	1st order exponential	0.05	N/A	1.06	N/A
43QP-GFP yeast [RNQ$^+$]	1st order exponential	0.04	N/A	1.30	N/A
97QP-EOS yeast dim	1st order exponential	0.50	N/A	0.45	N/A
97QP-EOS yeast bright	1st order exponential	0.03	N/A	11.04	N/A
43QP-GFP yeast dim (dual assemblies)	2nd order exponential	168.8	-0.0003	-52.88	-0.04
25QP-GFP *in vitro* droplet middle	1st order exponential	0.46	N/A	0.19	N/A
25QP-GFP *in vitro* droplet edge	1st order exponential	0.22	N/A	0.40	N/A

1st order exponential: $f(x) = A_1(1 - e^{(-\tau_1 x)})$

2nd order exponential: $f(x) = A_1\left(1 - e^{(-\tau_1 x)}\right) + A_2(1 - e^{(-\tau_2 x)})$

Table S2 FRAP instantaneous rates of recovery for data in Figure 2E.

Sample	Equation	p_1	p_2
43QP-GFP intra-assembly	1st order polynomial	116.8	6.11
43QP-GFP inter-assembly	1st order polynomial	2.13	115.0

1st order polynomial: $f(x) = \mathrm{p}_1 x + \mathrm{p}_2$

Instantaneous rates of recovery for a dim assembly due to (1) movement of 43QP-GFP within the assembly, and (2) transfer from a second dim assembly in the same cell, were obtained by fitting straight lines to the first few data points immediately after bleaching, or 2.6s after bleaching, respectively. Movement of 43QP-GFP is approximately 55-fold slower between assemblies than within individual assemblies.
